# Unexpected seasonality in quantity and composition of Amazon rainforest air reactivity

**DOI:** 10.1038/ncomms10383

**Published:** 2016-01-22

**Authors:** A. C. Nölscher, A. M. Yañez-Serrano, S. Wolff, A. Carioca de Araujo, J. V. Lavrič, J. Kesselmeier, J. Williams

**Affiliations:** 1 Air Chemistry and Biogeochemistry Department, Max Planck Institute for Chemistry, Mainz 55128, Germany; 2 Clima e Ambiente (CLIAMB), Instituto Nacional de Pesquisas da Amazônia (INPA), Avenue André Araújo 2936, Manaus, Amazonas CEP 69083-000, Brazil; 3 Embrapa Amazônia Oriental, Empresa Brasileira de Pesquisa Agropecuaria, Belem, Pará CEP 66095-100, Brazil; 4 Biogeochemical Systems Department, Max Planck Institute for Biogeochemistry, Jena 07745, Germany

## Abstract

The hydroxyl radical (OH) removes most atmospheric pollutants from air. The loss frequency of OH radicals due to the combined effect of all gas-phase OH reactive species is a measureable quantity termed total OH reactivity. Here we present total OH reactivity observations in pristine Amazon rainforest air, as a function of season, time-of-day and height (0–80 m). Total OH reactivity is low during wet (10 s^−1^) and high during dry season (62 s^−1^). Comparison to individually measured trace gases reveals strong variation in unaccounted for OH reactivity, from 5 to 15% missing in wet-season afternoons to mostly unknown (average 79%) during dry season. During dry-season afternoons isoprene, considered the dominant reagent with OH in rainforests, only accounts for ∼20% of the total OH reactivity. Vertical profiles of OH reactivity are shaped by biogenic emissions, photochemistry and turbulent mixing. The rainforest floor was identified as a significant but poorly characterized source of OH reactivity.

## Abstract (web summary)

The degree to which biogenic volatile organic compounds released by the Amazon canopy impact oxidation capacity remains uncertain. Here, the authors evaluate the vertical distribution of total hydroxyl radical reactivity and individual trace gases in the Amazon rainforest, and determine seasonal variations.

## Graphical abstract

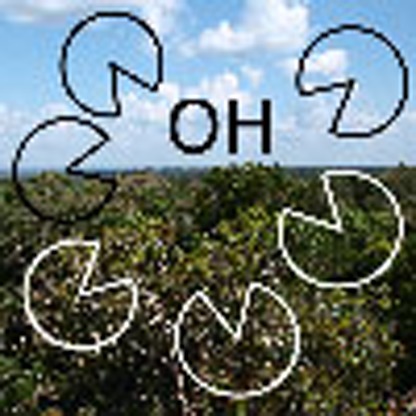

The Amazon rainforest is the largest, most productive, biodiverse, contiguous terrestrial ecosystem on Earth (about 6 million km^2^)^1^ and of global importance in terms of carbon, water and energy fluxes^2^,^3^,^4^. Its canopy delineates one of the most important interfaces between the terrestrial biosphere and atmosphere. This is where a complex mixture of highly reactive biogenic volatile organic compounds (BVOC) is released to the atmosphere^5^, among them isoprene, monoterpenes, other isoprenoids and various oxygenated compounds. Plant metabolic processes induce BVOC emissions that depend on light, temperature, stress, ambient concentration levels, water, nutrient availability, soil type, plant species and phenology^6^,^7^,^8^,^9^,^10^. After entering the atmosphere, BVOC are oxidized primarily by the hydroxyl (OH) radical—typically within seconds to hours. Therefore BVOC have a direct impact on ambient OH radical concentrations, or in other words the regional oxidation capacity of the atmosphere^11^. Since many greenhouse gases (for example, methane, CH_4_) and pollutants (for example, carbon monoxide, CO) are predominately oxidized in the Tropics by OH, variations of OH levels in these regions may have global scale consequences. The oxidation of BVOC by OH leads to a multitude of photochemical products that often remain undetected^12^,^13^. Recently, it has been found that some of these compounds partition to and from surfaces^9^,^14^,^15^ or participate in aerosol formation and growth^16^,^17^,^18^. Currently there are large uncertainties in how tropical aerosol influences the Earth's energy balance by directly effecting radiation or by modifying cloud characteristics^19^.

The combined effect of all OH reactive species on the instantaneous atmospheric OH loss frequency (in s^−1^) is defined as total OH reactivity, the inverse of the chemical lifetime of OH. It is dependent on the atmospheric concentration of all OH reactive trace gases [*X*_i_] and their reaction rate coefficient with OH (*k*_OH+*X*i_).


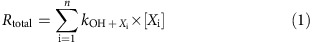


When total OH reactivity measurements are compared with individually detected BVOC levels, the relative contribution of each BVOC to the total OH reactivity can be determined. Moreover, the extent of missing or unaccounted for OH sinks can be evaluated. Previous studies in forested regions revealed a significant gap between the budget of individually detected known OH sinks and the directly measured total OH reactivity^13^,^20^,^21^. The missing reactivity has been shown to be dependent on the ecosystem, season, time of the day and environmental conditions. These unknown and undetected atmospheric OH sinks are likely to be either forest emissions^20^,^22^, the oxidation products of such^13^,^23^, or a complex mixture of both^21^,^24^. High missing OH reactivity implies an incomplete understanding of the processes occurring at the forest canopy–atmosphere interface such as biogenic emissions, gas-to-particle partitioning of oxidation products, atmospheric ozone formation potential, trace gas deposition inside a forest canopy, canopy dynamics, and the photooxidation of global pollutants and greenhouse gases.

Here we present the first detailed characterization of total OH reactivity seasonality, diel variation and vertical distribution throughout the canopy of a pristine Amazon rainforest. The results reveal how total OH reactivity is strongly influenced by biogenic emissions or uptake, atmospheric chemistry and physical mixing processes. Finally, we assess the composition and chemical nature of the total atmospheric OH reactivity through comparison with co-measured volatile organic compounds (VOC).

## Results

### Observations at the Amazonian tall tower observatory

Measurements were conducted in Central Amazonia within the framework of the Brazilian-German ATTO (Amazonian Tall Tower Observatory) project^25^. The measurement site is located about 150 km north-east of Manaus (120 m above sea level) in a dense, non-flooded upland forest. The immediate surroundings as well as large areas to the north-east, upwind of the site, are covered by an old-growth undisturbed forest, which permits natural process studies with minimal anthropogenic impact. Meteorological conditions at the ATTO site vary through the year according to the position of the inter-tropical convergence zone, with a cooler rainy season (February–May) and a hotter drier season (June–October)^26^.

Observations of greenhouse gases, reactive trace gases and aerosol within and above the forest canopy were conducted from an 80-m walk-up (INSTANT)^25^ tower (S02°08′38.8′′, W58°59′59.5′′). Micrometeorological conditions were continuously monitored and phenological observations were performed regularly. Since early 2012, this comprehensive set of measurements has served to characterize the tropical biosphere–atmosphere interface of the ATTO site^25^. Within this framework, total OH reactivity measurements were conducted by the comparative reactivity method (CRM)^27^,^28^, which is based on the competitive reaction of OH with a non-atmospheric molecule and all atmospheric OH sink compounds. In parallel, a quadrupole proton transfer reaction-mass spectrometer (PTR-MS) provided on-line measurements of various VOC^29^. Occasionally, air samples were collected on absorbent tubes for offline analysis of selected VOC in the laboratory using gas chromatographic methods.

Air for analysis was drawn down at ca. 16 l min^−1^ through eight continuously flushed, heated and insulated PTFE inlet lines (3/8′′=0.95 cm outer diameter) from the forest floor (0.05, 0.5 m), throughout understory and canopy (4, 12, 24, 38.3 m ≈average top of canopy), up to far above the canopy (53, 79.3 m). A valve system switched sequentially between the eight levels, completing one entire profile from 0.05 to 79.3 m in 16 min (Supplementary Fig. 1). Parallel to the total OH reactivity measurements, VOC (methanol, acetonitrile, acetaldehyde, acetone, isoprene, isoprene oxidation products, methyl ethyl ketone (MEK), benzene, toluene and the sum of monoterpenes), reactive trace gases (ozone (O_3_), nitric oxide (NO), nitrogen dioxide (NO_2_)) and carbon dioxide (CO_2_)) were sampled via the same inlet system. The inlet system was tested with a gas standard for all detected VOC and no significant differences between the various heights were observed. The line losses (difference between expected and detected VOC mixing ratios) were small, on average 9% (see Methods).

### Seasonal variation

Four intensive field measurement campaigns were performed over a 1-year period from October 2012 to September 2013 at the ATTO site (Supplementary Fig. 2).

**Figure 1:**
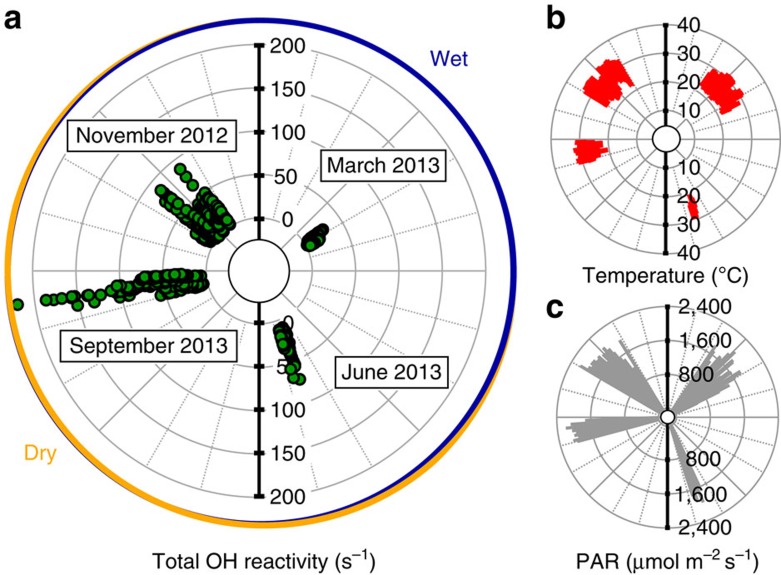
One year of total OH reactivity measurements in Amazon rainforest air. Overview of total OH reactivity canopy observations at 24 m (**a**), temperature at 26 m (**b**) and photosynthetically active radiation (PAR) at 75 m (**c**) during four measurement campaigns during the dry-to-wet-season transition period (November 2012), the wet season (March 2013), the wet-to-dry-season transition period (June 2013) and the dry season (September 2013).

The first observations were made in the transition period between dry and wet season (31 October 2012–29 November 2012). Typically, the days were hot, reaching maximum values inside the forest canopy of up to 35 °C. Photosynthetically active radiation (PAR) was highly variable due to isolated convective clouds moving overhead and occasional rain events (Fig. 1, Table 1). Significant diel variability of total OH reactivity was found ranging from the instrument's detection limit (3–4 s^−1^) to 135 s^−1^ (observed at the 79.3 m level in the afternoon). Inside the canopy (24 m) and above the canopy (79.3 m) comparable averages of 20.7±16.8 and 19.6±14.2 s^−1^ (campaign mean±s.d.) were determined, respectively.

During the colder, generally overcast and rainier wet season measurement campaign (27 February 2013–6 March 2013) total OH reactivity decreased to about half of what was observed during the dry-to-wet-season transition period, to on average 9.9±5.2 s^−1^ inside the canopy at 24 m. These levels of total OH reactivity are, for example, comparable to the typical summertime boreal forest (no prolonged heat, no anthropogenic or biomass burning influence)^21^,^30^.

In the wet-to-dry-season transition period (8–14 June 2013), average temperature and radiation increased relative to the previous campaign. The total OH reactivity rose inside the forest canopy to on average 26.1±13.8 s^−1^. This was higher than the average canopy total OH reactivity observed during the dry-to-wet-season transition. Comparable total OH reactivity levels and variability were recently observed in a Mediterranean Oak forest which copiously emitted isoprene^31^.

At the midpoint of the dry season (20–29 September 2013), days were typically dry and hot with overall highest annual midday averages for PAR (Table 1). At that time of the year, total OH reactivity reached maximum values up to 241 s^−1^ inside the Amazon rainforest's canopy in the afternoon of 23 September 2013 (Supplementary Fig. 2). Similar high total OH reactivity has been reported previously only in the extremely polluted environment of Mexico City at rush hour^32^. Overall, this was the season with the largest average total OH reactivity of 62.4±41.3 s^−1^ within the canopy.

### Diel variation

**Figure 2:**
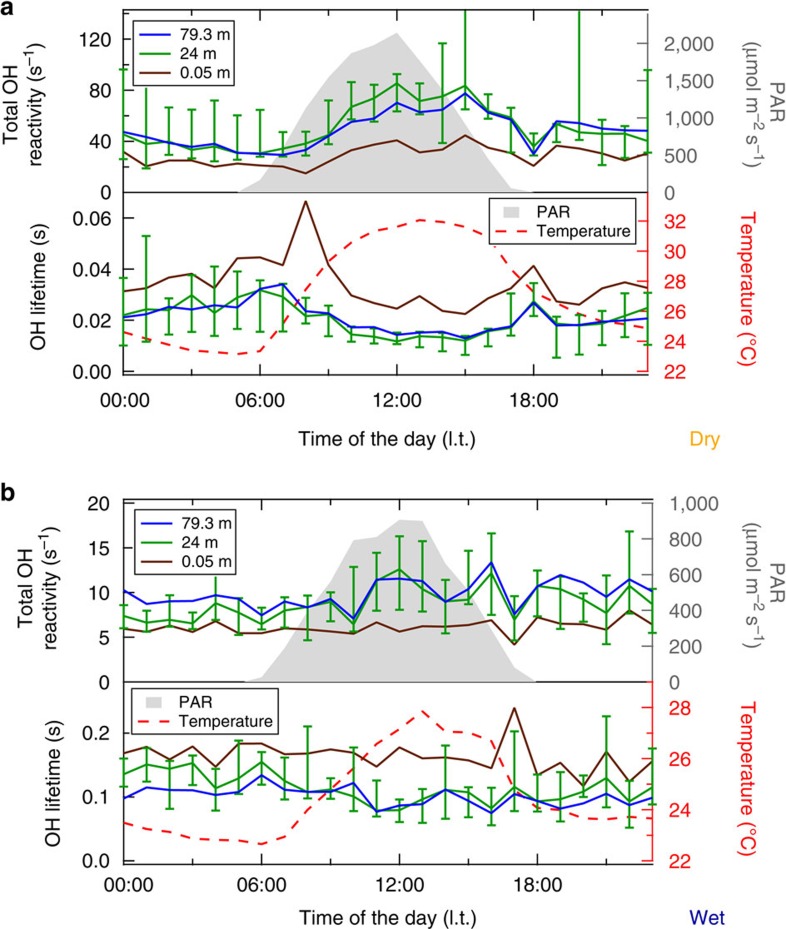
Dry and wet season total OH reactivity and OH chemical lifetime. Figure panels (**a**) and (**b**) show soil (0.05 m), canopy (24 m) and atmospheric (79.3 m) total OH reactivity and OH chemical lifetime during dry (September 2013) and wet season (March 2013), respectively. Diel hourly medians are plotted as function of the time of the day, with ambient temperature (at 26 m) and the photosynthetically active radiation (PAR). Exemplarily the ±75/25 percentiles are pictured as error bars for the 24 m total OH reactivity (OH lifetime) medians.

Figure 2 presents diel hourly medians of the total OH reactivity and its inverse, the hydroxyl radical chemical lifetime. Independent of the season, sunrise and sunset were at approximately 06:00 and 18:00 l.t. (local time) and the peak of radiation was at noon. The diurnal peak in ambient temperature was shifted compared with PAR, reaching its maximum in the afternoon at 13:00 l.t. for the wet season and 15:00 l.t. for the dry season.

The scales for total OH reactivity in Fig. 2 differ by about a factor of 7 with significantly higher median values in the dry season. Total OH reactivity ranged between 6 and 13 s^−1^ within the canopy at 24 m for the wet season. Clear diel trends were observed for the dry season with median total OH reactivity inside the canopy varying between 31 and 85 s^−1^. Maximum values were found during daytime, decreasing throughout the night. For both seasons the weakest diel variation and the lowest absolute values of total OH reactivity were found at the lowest level, 0.05 m from the soil. During the dry season, the median total OH reactivity inside the canopy (24 m) reached peak values with maximum PAR at noon and maximum temperature in the afternoon. This is in close accordance with previous studies^5^,^7^,^33^ that identified light and temperature as the major driving forces for biogenic emissions.

The inverse of the total OH reactivity measurements directly provides the OH chemical lifetime. It is determined by all instantaneous OH loss reactions at each observed level within and above the Amazon rainforest. The dry-season median OH chemical lifetime inside the canopy in the afternoon was extremely short and reached minimum values of <10 ms, while during the wet season it became an order of magnitude longer (100–200 ms).

### Vertical variation

**Figure 3:**
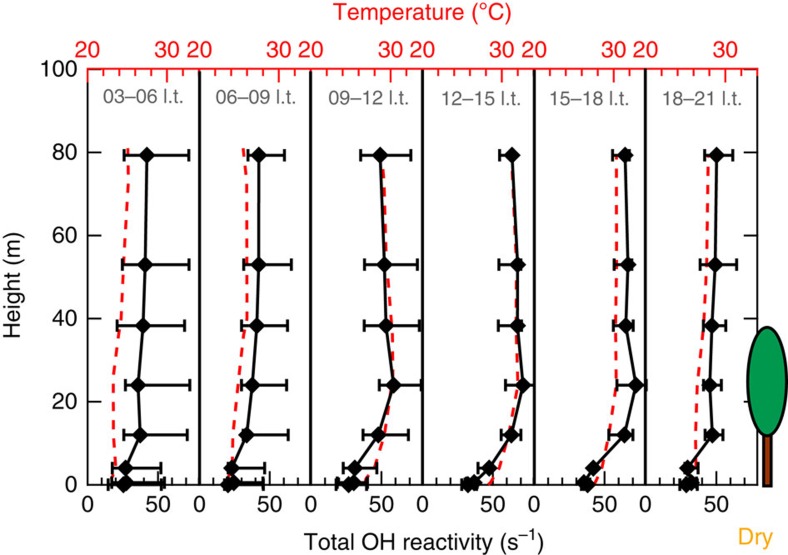
Dry-season vertical profiles of total OH reactivity and temperature between forest floor and 79.3 m. Six different profiles present the temporal variation throughout the day for 3 h-binned median total OH reactivity and ambient temperature. Positive error bars include the 75 percentiles of the dry-season campaign (September 2013) total OH reactivity measurements, negative error bars the 25 percentiles.

Before sunrise (03:00–06:00 l.t., September 2013) the dry-season median total OH reactivity (35.8–42.2 s^−1^) stayed rather constant throughout understory, canopy and atmosphere above the canopy (12–79.3 m, Fig. 3). At that time the ambient temperature (23.0–25.1 °C) did not vary significantly from soil to uppermost level. At sunrise vertical gradients were smallest and total OH reactivity at its diel minimum (Fig. 2). This was when the shallow nocturnal boundary layer started to break up. Aged and less-reactive air from higher altitudes was likely mixed down and diluted the rainforest reactive conglomerate^34^.

With the onset of solar irradiation, the forest canopy began to heat up. Already by 09:00–12:00 l.t. the canopy temperature was significantly enhanced. With rising irradiation and temperature, the total OH reactivity increased strongly to maximum levels inside the canopy. A strong temperature inversion at 4 m isolated the lower cooler layer dynamically throughout the entire day which led to minimum total OH reactivities between 20 and 40 s^−1^ (Fig. 3). Interestingly, surface soil temperature (23.7–26.2 °C) and water content (0.16–0.27) did not vary significantly, neither during the day nor throughout the tropical seasons (Supplementary Fig. 3). During the dry-season day (12:00–15:00 l.t.) the total OH reactivity developed a strong gradient, decreasing rapidly from 71.5 s^−1^ (3 h median, 24 m) towards the soil level to 32.3 s^−1^ (3 h median, 0.05 m) and decreasing more moderately above the canopy to 63.7 s^−1^ (3 h median, 79.3 m). This development reflects the emission source location (canopy), the rate of oxidation of reactive primary emissions to less-reactive products (above canopy) and the turbulent exchange rates at each height (mixing above the canopy, stratification below the canopy).

**Figure 4:**
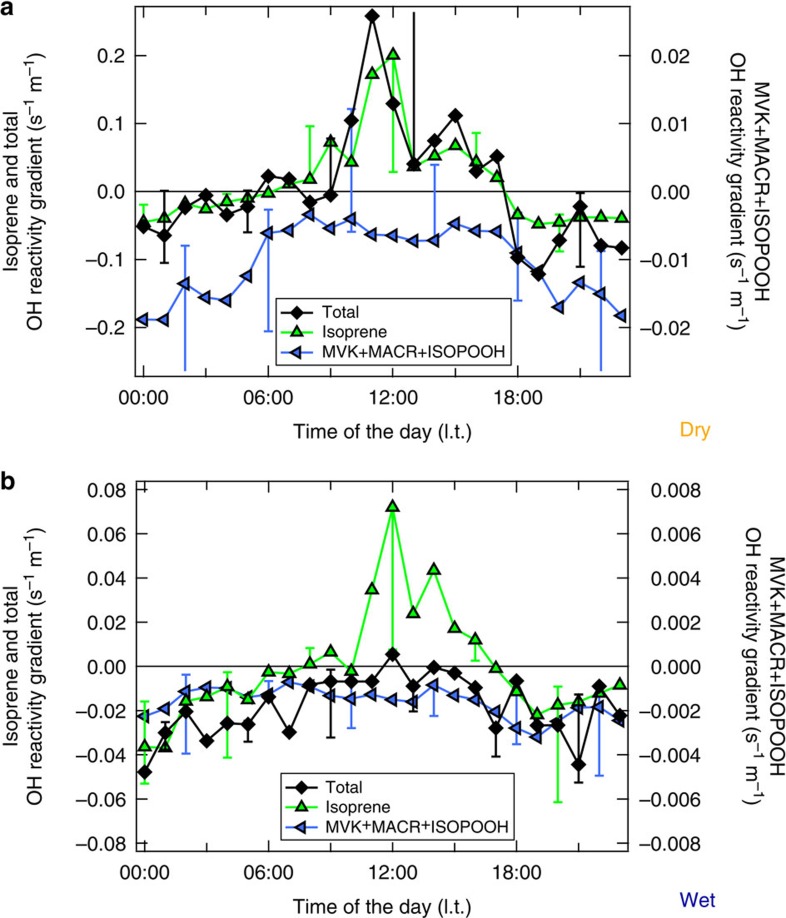
Dry and wet season diurnal variation of OH reactivity median gradients above the canopy. The gradients were calculated as linear regression slope for the total OH reactivity, isoprene OH reactivity and isoprene products (MVK+MACR+ISOPOOH) OH reactivity above the canopy. A gradient equal to zero corresponds to constant OH reactivity with height. A negative gradient goes along with higher values in 79.3 m, and a positive gradient with higher values inside the canopy (24 m). Median OH reactivity gradients above the canopy were calculated for dry (**a**) and wet (**b**) season measurement campaigns in September 2013 and March 2013. Error bars picture the variability of the median OH reactivity gradients above the canopy as the 75-(positive) and 25-(negative) percentiles.

Figure 4 compares the relative direction of vertical gradients above the canopy as function of season and time of the day for the total OH reactivity and two prominent atmospheric reactants with OH. Isoprene is copiously emitted by tropical vegetation and is highly reactive (*k*_OH+ISO_(298 K)=1 × 10^−10^ cm^3^ molecule^−1^ s^−1^, IUPAC)^16^,^35^. Prominent oxidation products from the reaction of isoprene and OH^12^ are methyl vinyl ketone (MVK), methacrolein (MACR) and isoprene hydroxy hydroperoxides (ISOPOOH) whose summed signal is detected by the PTR-MS (*m*/*z* 71)^36^,^37^. Detected atmospheric isoprene and isoprene product (MVK+MACR+ISOPOOH) mixing ratios were used to determine the OH reactivity of isoprene (representing light and temperature-dependent primary emissions) and of isoprene products (representing photochemistry products). Total, isoprene and MVK+MACR+ISOPOOH OH reactivities were plotted as a function of height in and above the canopy (24, 38.3, 53, 79.3 m) and a linear regression slope was calculated (Supplementary Fig. 4). Gradients above the canopy were positive when OH reactivity was greater inside the canopy (24 m), and negative when OH reactivity was greater at the top levels (79.3 m).

Wet-season vertical gradients relative to the canopy were less pronounced than in the dry season. However, the night-to-daytime variability of isoprene and MVK+MACR+ISOPOOH OH reactivity gradients was consistent throughout the seasons. Both compounds had negative gradients during the night (higher levels at 79.3 m). After sunrise isoprene was released from the forest canopy (positive gradients), whereas MVK+MACR+ISOPOOH were photochemically produced above the canopy (negative gradients). Unlike these two classes of important OH sinks (emissions and oxidation products), the total OH reactivity vertical distribution unexpectedly changed its diurnal pattern with the season. During the wet season, total OH reactivity gradients were mostly negative and close to zero, similar to the observed isoprene products. Due to the relatively slow reaction with OH (*k*_OH+MVK+MACR+ISOPOOH_(298 K)=5.62 × 10^−11^ cm^3^ molecule^−1^ s^−1^, average MVK+MACR+ISOPOOH rate coefficient, IUPAC, MCM v3.3), the absolute gradient of MVK+MACR+ISOPOOH OH reactivity was about a factor of 10 smaller than the total OH reactivity. During the dry season, total OH reactivity gradients matched isoprene, being positive by day and negative at night. This shows that the vertical distribution of predominant OH sinks strongly depends on the tropical season. Photooxidation products, such as MVK+MACR+ISOPOOH-like compounds, shape the vertical gradient of the wet season total OH reactivity. These species persist and can be transported further above the canopy due to their longer lifetimes. Within the canopy they can be efficiently uptaken by the wet surfaces decreasing their OH sink potential. In the dry season, isoprene-like compounds dominate the total OH reactivity vertical distribution. The significantly enhanced total OH reactivity in the dry-season canopy layer was potentially caused by strong biogenic emissions of reactive compounds due to increased PAR and leaf temperatures, *in situ* oxidation by strong irradiation reaching inside the canopy, and the reduced deposition due to drier surfaces, through less-opened stomata^38^, and therefore a smaller leaf area^39^. Another possibility is that microbial communities on leaf surfaces modulate ambient BVOC levels^40^,^41^ due to seasonal variations in number and species.

### Missing reactivity

**Figure 5:**
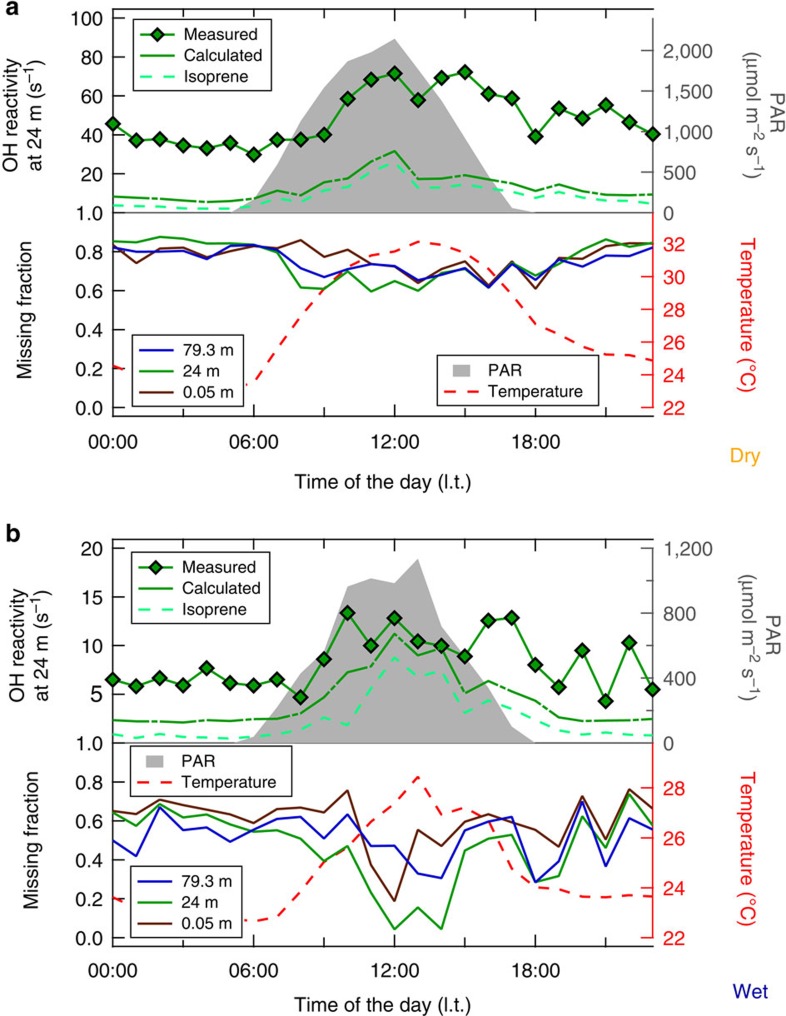
Comparison of measured and calculated OH reactivity for dry and wet season. Dry (**a**) and wet (**b**) season OH reactivity was measured, and calculated due to isoprene alone and all co-detected reactive compounds. The relative difference between measured and calculated OH reactivity is the missing fraction, the ratio of unmeasured OH sinks to the total measured OH reactivity. The missing fraction is presented for three levels: soil (0.05 m), canopy (24 m) and top of the tower (79.3 m). Total OH reactivity hourly medians slightly differ from Fig. 2, because only data with full coverage of all measured compounds have been considered in the calculation. For comparison, PAR and temperature diel hourly medians were included in this figure.

The atmospheric OH reactivity was calculated as the sum of all individually detected OH sinks: methanol, acetonitrile, acetaldehyde, acetone, isoprene, MVK+MACR+ISOPOOH, MEK, monoterpenes, benzene, toluene, CO, NO, NO_2_, O_3_ and CH_4_ (Supplementary Note 1; Supplementary Table 1; Supplementary Fig. 5). Figure 5 presents a comparison of the diurnal variation for wet- and dry-season OH reactivity—calculated, measured and missing. The gap between calculated and measured total OH reactivity was significant and variable as a function of season, time of the day and height. On average, 49±24% (4.1±3.0 s^−1^) of the measured total OH reactivity inside the canopy remained unaccounted for during the wet-season measurement campaign (March 2013). At midday most of the total OH reactivity within the forest canopy could be explained with only 5–15% missing, whereas below and above the forest canopy this fraction was greater. The dry season missing reactivity was greater with on average 79±11% (51.5±29.4 s^−1^) in 24 m. Even though isoprene was the strongest OH sink among the detected VOC, it only accounted for about one-fifth of the total OH reactivity inside the canopy during the dry-season afternoon. For both seasons the measured total OH reactivity could be better explained during the daytime inside the canopy when isoprene emissions were high.

**Figure 6:**
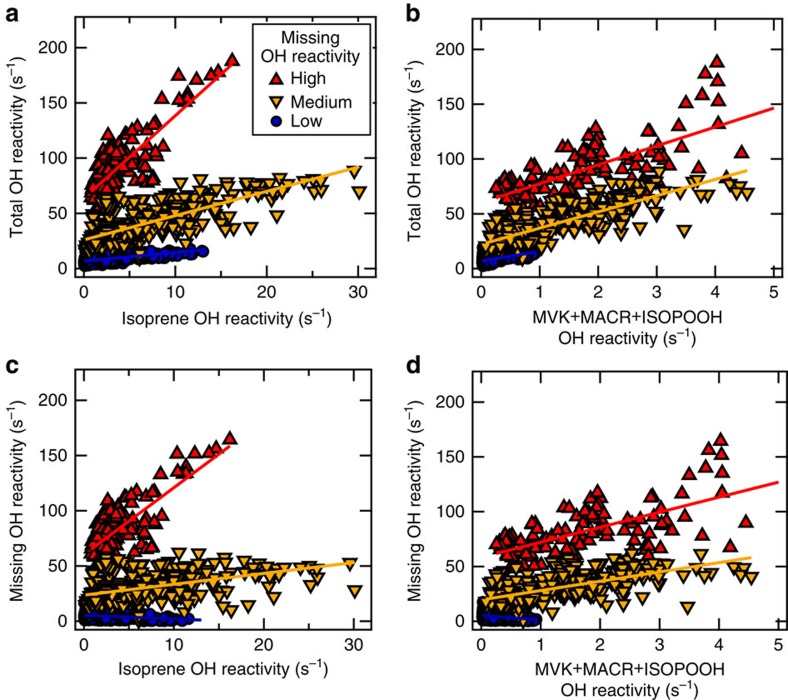
Total or missing OH reactivity correlations with isoprene or MVK+MACR+ISOPOOH OH reactivity. For low (wet season), medium (dry season, <60 s^−1^) and high (dry season, >60 s^−1^) missing OH reactivity, total OH reactivity was correlated with isoprene and its oxidation product's MVK+MACR+ISOPOOH OH reactivity in (**a**) and (**b**), and missing OH reactivity was correlated with isoprene and MVK+MACR+ISOPOOH OH reactivity in (**c**) and (**d**).

High missing OH reactivity has been previously observed in forested environments and explained as either forest primary emissions^20^,^22^ or photooxidation products of such^13^,^23^ or a mixture of both^21^,^24^. In the Amazon rainforest the most prominent OH sinks are isoprene, emitted by trees with high light and temperature, and its oxidation products, formed above the canopy in the sunlit atmosphere. Total and missing OH reactivity are weakly, but positively correlated to the observed isoprene and MVK+MACR+ISOPOOH OH reactivities (Supplementary Fig. 6). Figure 6 and Table 2 show that this correlation can be partitioned according to the level of missing OH reactivity: Extremely high missing OH reactivity (>60 s^−1^) that occurred during dry-season nights (no daytime observations available); medium missing OH reactivity (<60 s^−1^) during several days at the end of the September 2013 dry-season campaign; and low missing OH reactivity (<20 s^−1^) during the wet season (March 2013).

Dry season total OH reactivity correlated best with isoprene and its oxidation products (0.74<Pearson correlation coefficient (Pearson correlation coefficient (PCC))<0.82) for both high and medium missing reactivity, whereas correlations were worse for the wet-season observations in times of low missing reactivity (0.49<PCC<0.50) (Table 2). For all periods, missing OH reactivity correlated not as well as the total OH reactivity with isoprene or its measured oxidation products (MVK+MACR+ISOPOOH). Missing OH reactivity during the wet season (low missing reactivity) was even anti-correlated to isoprene and its oxidation products (PCC=−0.27).

The slope between total OH reactivity and isoprene OH reactivity equals 2.2±0.1 for medium missing reactivity, which is similar to the value reported by Edwards *et al*.^13^ for the Borneo tropical rainforest (measured at a height of ∼5 m). In Amazon rainforest air, the slope is higher (7.5±0.5) for high missing OH reactivity and lower (0.8±0.04) for low missing OH reactivity during the wet season. Edwards *et al*.^13^ contend that higher slopes (up to 10) indicate low physical losses or higher OH, whereas smaller slopes are caused by enhanced physical loss.

**Figure 7:**
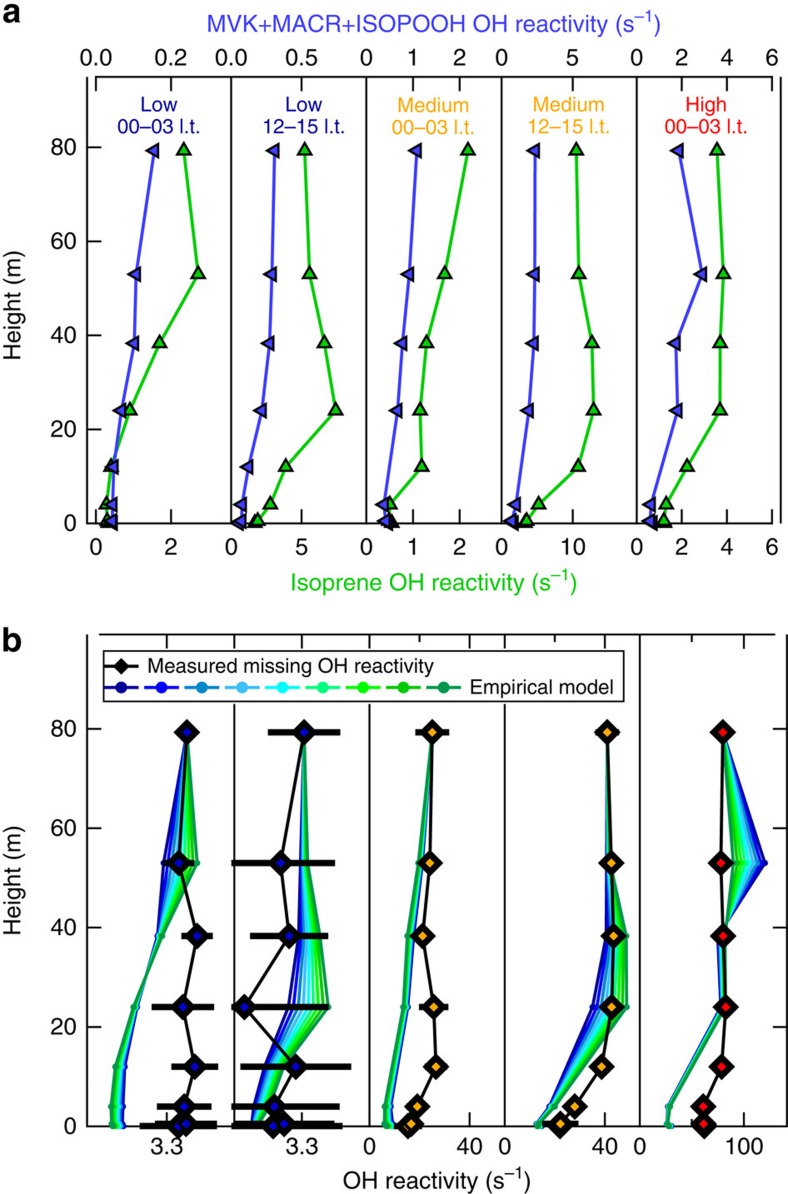
Median vertical profiles for night and day during three regimes of missing OH reactivity. (**a**) Isoprene and isoprene products (MVK+MACR+ISOPOOH) OH reactivity. (**b**) Missing OH reactivity (markers with uncertainties ±95 percentiles) and empirical model that is calculated as the sum of isoprene and isoprene products OH reactivity multitudes. To account for the missing OH reactivity in 79.3 m the mixture of typical biogenic emissions (isoprene) and typical photooxidation products (MVK+MACR+ISOPOOH) was varied in ratios from 90:10 to 10:90, and the resulting typical vertical profile (empirical model) was coloured green for an emission dominance and blue for a products dominance.

Whether the behaviour of the missing OH reactivity is more like biogenic forest emissions or atmospheric photooxidation products is examined in Fig. 7. Median vertical profiles for isoprene, detected isoprene oxidation products and missing OH reactivity were calculated for night (00:00–03:00 l.t.) and day (12:00–15:00 l.t.) during low, medium and high missing reactivity periods. Similar to Fig. 4, it can be seen that MVK+MACR+ISOPOOH typically reached highest values at the top level (79.3 m) where oxidation, mixing and transport dominate the atmospheric composition, and that isoprene typical profiles build up maximum levels during daytime inside the canopy (24 m) following the diurnal cycle of light- and temperature-driven forest emissions (Fig. 7, top panel). In Fig. 7 (bottom panel), we apply an empirical model to elucidate missing OH reactivity. The entire missing OH reactivity determined at 79.3 m was assumed to consist of a mixture of unknown biogenic tree emissions and undetected oxidation products. For example, in the case of the dry-season daytime medium missing reactivity, the missing OH reactivity at the top level was 41 s^−1^. If this missing OH reactivity is attributed to 50% emissions and 50% oxidation products, then the mixture would have had to be as large as two times the observed isoprene and almost nine times the MVK+MACR+ISOPOOH reactivities. Within the framework of this empirical model, the ratio of isoprene to isoprene oxidation products was varied (from 90:10 to 10:90) to account for the 79.3 m missing OH reactivity. This way, the typical vertical profiles of isoprene and MVK+MACR+ISOPOOH reactivities were scaled and summed, while varying between plausible extremes in primary versus products mixtures (Supplementary Note 2; Supplementary Table 2). Therefore, we can map out the span of possible vertical profiles: from being shaped like biogenic tree emissions (isoprene-like) to photooxidation products (MVK+MACR+ISOPOOH-like; Fig. 7, bottom panel).

Daytime medium missing OH reactivity vertical gradients were well reproduced in the empirical model by the mixture of biogenic emissions and photooxidation products inside and above the forest canopy. Below the canopy the model decreased faster towards the soil than the measured missing OH reactivity profile. This difference is depicted as a residual greater than zero in Supplementary Fig. 7. Close to the ground this gap persists for all analysed median profiles—independent of the season. Especially during nighttime, the typical profiles of tree emissions (isoprene) and (isoprene) photooxidation products fail to explain the vertical distribution of missing OH reactivity. In addition, during wet-season days, observed missing OH reactivity and model profiles show opposite tendencies, which is consistent with the wet-season anti-correlation between missing OH reactivity and isoprene or isoprene products (Fig. 6).

The assumption that the Amazon rainforest missing OH reactivity consists of isoprene-like and isoprene oxidation products-like compounds simplifies the question about the source of unknown and unmeasured OH sinks. Applying this assumption within a simple empirical model has revealed that the vertical distribution of missing OH reactivity cannot be explained by the sum of biogenic tree emissions peaking inside the canopy and photooxidation products formed above the canopy alone. Additional sources of OH reactive compounds need to be considered from lower down, potentially in the understory vegetation, the forest floor or soil.

### Potential candidates for missing reactivity

If the average dry-season missing OH reactivity inside the canopy (51.5±29.4 s^−1^) was due to a single compound (*k*_OH+*X*_=5 × 10^−11^ cm^3^ molecule^−1^ s^−1^), then this compounds mixing ratio would need to be over 40 p.p.b.V. (parts per billion by volume). This mixing ratio is greater than that of isoprene and would not likely have remained undetected by our measurement systems. More likely is that a myriad of primary and secondary VOC are present at substantially lower mixing ratios.

Decaying plant material, soil or litter emissions can provide additional sources of VOC and thereby OH reactivity. Isidorov *et al*.^42^ reported 77 species emitted from microbiologically decomposed leaf litter of 5 different deciduous trees. For 59 of those compounds a rate coefficient with OH is reported in the literature. Their average rate coefficient (*k*_OH+LITTER_=3.22 × 10^−11^ cm^3^ molecule^−1^ s^−1^) was applied for compounds with no literature value available. Excluding the compounds that were determined during our measurements and hence are already included in the OH reactivity budget, the calculated OH reactivity of typical litter emissions is 2.8 s^−1^. This assumes that all of these compounds were present in the atmosphere with levels of 100 p.p.t.V. Clearly higher mixing ratios could be present for many of these species. Some samples of Amazon rainforest air that were analysed offline with a gas chromatograph-flame ionization detector (GC-FID) had hexane in mixing ratios up to 700 p.p.t.V. Hexane was part of the Isidorov *et al*.^42^ list along with methylfuran, 2-butene and ethyl mercaptan, all of which are very reactive to OH. Likely, the tropical soil and leaf litter emitted a broader variety of compounds than the leaf litter of the five different deciduous trees examined in Isidorov *et al*.^42^. Overall, the tropical forest floor seems to be a potentially important but as yet insufficiently characterized source of OH reactivity.

Physical damage and herbivory elicits specific stress emissions from vegetation that can also contribute to reactivity, especially in the lower canopy and understory where light- and temperature-driven emissions decrease. These include benzenoid compounds that have been recently reported by Misztal *et al*.^43^. However, even assuming 100 p.p.t.V. of the 27 compounds by Misztal *et al*.^43^ leads to only about 2 s^−1^ OH reactivity. This calculation includes as an approximation for missing rate coefficients the average rate coefficient of the 14 known rate constants (*k*_OH+BENZENOID_=2.67 × 10^−11^ cm^3^ molecule^−1^ s^−1^). Benzene and toluene have been excluded as OH sinks because these two compounds were already part of the calculated OH reactivity. It has to be noted that the benzenoid compounds may represent only a fraction of stress-induced biogenic emissions, and that the photochemical products of these species are often more reactive than the emissions themselves^44^.

Courtois *et al*.^45^ measured 264 and identified 206 stress-related VOC from tropical forest in French Guiana using gas chromatographic-mass spectrometry (GC-MS). Methyl salicylate, hexanal, linalool and many highly reactive sesquiterpenes are part of this budget. The 33 known literature reaction rate coefficients with OH were averaged (*k*_OH+TROPICAL_=8.5 × 10^−11^ cm^3^ molecule^−1^ s^−1^) and used for all VOC without known rates. As monoterpenes were measured and already were part of the calculated OH reactivity, they were excluded at this point. For atmospheric levels of 100 p.p.t.V., we estimate the OH reactivity of 36.1 s^−1^ that can be produced from these stress-related species. This is in the order of missing OH reactivity observed in the dry-season Amazon rainforest.

In fact, there are thousands, if not tens of thousands of such reactive organic compounds in the atmosphere^46^ emitted from the biosphere and oxidized to numerous products in the air. Many of these compounds are present in relatively low concentrations, below the detection limit of many instruments, or not detected by currently available field equipment. However, their summed effect is visible in the total OH reactivity, as ozone production potential, or as a trigger for aerosol formation and growth.

## Discussion

Important driving forces of total OH reactivity at the ATTO site were identified to be of biological (BVOC emissions), chemical (oxidation of BVOC) and physical (deposition, turbulence and thermal stratification) nature.

The OH chemical lifetime inside the canopy varied enormously (by a factor of 10), between wet and dry season (Fig. 2). At the time of the shortest local, chemical OH lifetime, during dry season, a large fraction of the total OH reactivity remained unaccounted for (on average 79% at 24 m) despite isoprene and its main photooxidation products (MVK+MACR+ISOPOOH) being measured. In that season a good match between isoprene and the total OH reactivity vertical profiles above the canopy was observed (Fig. 4). The missing OH reactivity correlated well with both isoprene and its oxidation products in September 2013 (Fig. 6). Accordingly, the daytime missing OH reactivity gradient could be mimicked well by combining the typical biogenic emission profile of isoprene and the typical atmospheric oxidation product of isoprene photooxidation products (Fig. 7, medium missing reactivity). At those times, the missing OH reactivity was likely dominated by an as yet undefined mixture of biogenic emissions and photooxidation products. Potential candidates for these unmeasured species are forest emissions triggered by temperature and light or by mechanical and herbivoral stress, such as green leaf volatiles (C6 aldehydes and ketones), benzenoid compounds^43^, multifunctional entities containing an alkene bond (for example, hexenal, hexenol)^5^,^6^ or sesquiterpenes in very low levels. In addition, several product families of VOC photooxidation^13^,^47^,^48^, such as peroxides, aldehydes (for example, formaldehyde) and epoxides, that are challenging to determine during field campaigns, were not measured at the ATTO site.

For the time of the longest local, chemical OH lifetime, during wet season, missing OH reactivity was smallest. Vertical gradients of total OH reactivity matched those of photooxidation products (Fig. 4). The daytime total OH reacitivity within the canopy was mostly explained by co-measured OH sinks. However, the remaining missing OH reactivity gradient could not be accounted for by any combination of biogenic emissions and oxidation products (Figs 6 and 7), especially during nighttime and below the canopy. Overall, the less oxidative atmosphere, the relatively high deposition of oxidation products on wet surfaces^49^ and possible enhanced leaf surface microbial activity^40^,^41^ helped to close the total OH reactivity budget in the wet season.

Throughout all seasons the total OH reactivity was lowest at the forest floor, but interestingly the average missing OH reactivity was always high there (Fig. 5). The vertical gradients of missing OH reactivity in the lowermost layers of the rainforest could not be explained by the vertical distribution of biogenic tree emissions (isoprene-like), or by the profile of oxidation products (isoprene products-like), or by a combination of both (Fig. 7). A thermal inversion below the forest canopy hindered mixing between canopy and soil level (Fig. 3). Hence, soil, litter and understory vegetation likely affected the budget of reactive compounds in this lowermost layer through emission and deposition^5^,^50^. Compounds such as nitrous acid (HONO)^51^ and some VOC (for example, organic acids and multifunctional carbonyls)^5^,^50^ emitted as a result of microbial activities in soils and leaf litter were not measured in this study, but likely also contributed to the Amazon rainforest total OH reactivity near the forest floor.

The seasonal, diel and vertical variations of total and unaccounted for air reactivity have important implications and research challenges for several adjacent fields such as plant–plant and plant–insect interactions, microbiology, soil science, biogeochemistry and carbon cycling. High reactivity and therefore short OH lifetime can impact the effectiveness of insect pollination or the timing of windborne pollen dispersal. Microbial communities in soils and on leaves may play a very active, but as yet poorly understood role in regulating total OH reactivity at the biosphere–atmosphere interface. Their effect on atmospheric composition and oxidation potential was exposed in this study as a missing OH reactivity at soil level and postulated on leaf surfaces based on the wet-season data (negative total OH reactivity vertical gradients and minimum missing reactivity). Long-term observations of Amazon rainforest OH reactivity of the kind shown here also have the potential to reveal how the natural forest–atmosphere interface changes when under the influence of biomass burning, anthropogenic pollution or even El Niño events. Total OH reactivity can also be considered as a measure for carbon released by the tropical vegetation or soils weighted by its reaction potential with OH. Especially during the dry season the amount of OH reactive carbon in the Amazon rainforest air was substantial presenting a new challenge to carbon cycling models.

This comprehensive characterization of tropical rainforest total OH reactivity was achieved by the high temporal and vertical resolution of the total OH reactivity observations. The vertical gradients spanning eight measurement levels from the forest floor up to almost 80 m into the atmosphere have revealed unexpected variations in seasonal, diel and vertical total OH reactivity distribution, composition and strength, as well as a hitherto overlooked influence of the forest floor on OH reactivity.

## Methods

### Details about the ATTO measurement site

ATTO^25^ is embedded in undisturbed old-grown rainforest. The rainforest upwind of the site as well as in close distance is a species rich ‘terra firme' forest. Highest tree species richness was observed at the plateau adjacent to the 80 m tower. Here, over 400 species have been identified^25^. The average tree height was about 38 m. Observations from the 80 m walk-up tower of the leaf area index (LAI) showed a high variability with height and time. On average the LAI was 5.5 with timely variable biomass at different levels inside the canopy or in the understory (monitored with LICOR 2200, personal communication Giordane Martins). The understory surrounding the measurement tower is rich in small, young, growing trees, palms and ferns. Mosses and lichen frequently cover stems and leaves of old-grown trees. Leaf litter and other decaying plant material such as branches or fine roots cover densely the clumpy, red soil (Supplementary Fig. 1). Detailed information about forest phenology and soil characteristics at the ATTO site can be found in Andreae *et al*.^25^.

Since 2012, continuous meteorological observations are available for the ATTO site. The near-equatorial location defines two characteristic seasons depending on the inter-tropical convergence zone (ITCZ) and its associated wind systems^26^. As the ITCZ moves to the north, the main wind direction is east and southeast during the dry season (July–November). During this time potential influence from biomass burning or human activities from eastern Brazil may be observed at the site. As the ITCZ moves to the South, wet-season winds are directed from northeast (January–March). At that time, air masses pass over long distances of pristine and undisturbed rainforest before arriving at the ATTO site.

Continuously monitored are also aerosol parameters (that is, black carbon, aerosol scattering, particle number concentration, aerosol number size distribution, particle mass and chemical speciation), greenhouse gases (CO_2_, CH_4_) and other trace gases (CO, NO, NO_2_, O_3_). The VOC and total OH reactivity observations have been performed during intensive field measurement campaigns.

### Vertical gradient set-up

At the ATTO site an 80-m walk-up (INSTANT)^25^ tower was installed hosting an inlet system that allowed sampling air from eight different heights (0.05, 0.5, 4, 12, 24, 38.3, 53 and 79.3 m). The three lowermost inlets (0.05, 0.5 and 4 m) were mounted on a tripod in close proximity (12 m) to the 80-m walk-up tower but at a more undisturbed location inside the forest. The inlet lines were made of 3/8′′ (0.95 cm) diameter non-transparent PTFE tubing, they were heated, insolated and continuously flushed with a high flow. At the top of each line, the sample was passed through a regularly renewed PTFE filter (5 μm) to avoid impact of aerosol and insects. For sampling, a Teflon pump drew down air from all inlets with a flow rate of ∼16 l min^−1^.

Every 2 min a Teflon valve system switched to the next level, completing one entire profile from 0.05 to 79.3 m in 16 min. With this inlet system, vertical gradients of ozone (O_3_), nitric oxide (NO), nitrogen dioxide (NO_2_), carbon dioxide (CO_2_), VOC and total OH reactivity were monitored simultaneously. The equipment was hosted in a temperature-controlled laboratory container adjacent to the 80-m walk-up tower. The instruments were subsampling from the gradient system via short and insolated Teflon lines.

### Inlet line tests

The inlet lines of the gradient system have been tested for losses and variable response that could result from the different length of the tubing. The VOC calibration standard gas was used to spike into the lines while sampling ambient air at ground level (0.5 m), just below the canopy (12 m) and the top level (79.3 m). The typical 2 min switch cycle of the gradient measurements was kept and the ‘spiked' level was compared with the previous and next measurement point at the same height. The concentration levels of the spikes were chosen high enough to stay above ambient levels (12–16 p.p.b.V.). On average the line losses were about 9% varying between the different VOC. Most importantly we observed no significant differences between the three tested inlet lines.

Since the losses were minor, the inlet line response independent of the length and both instruments sampled in parallel from the same lines, no corrections were applied and the original data are presented.

### Total OH reactivity measurements

The total OH reactivity was directly measured using the CRM^21^,^27^. This method is based on a competitive reaction of OH with a reagent which is not usually present in ambient air, and OH with the atmospheric OH reactive compounds. We used an automated set-up for the CRM and a PTR-MS (operated in standard conditions: 2.2 mbar drift pressure, 600 V drift voltage and 127 Td) as detector for the reagent pyrrole (C_4_H_5_N) as described in Nölscher *et al*.^21^ Pyrrole was mixed inside a Teflon-coated glass reaction cell with zero air (dry) and nitrogen (dry) to obtain the initial concentration level of pyrrole [C1]. Then, nitrogen was humidified and flushed over a ultraviolet lamp to generate OH molecules that react inside the glass reactor with pyrrole. The detected pyrrole level [C2] dropped according to the generated amount of OH inside the reactor. During this step catalytically scrubbed air (Platinum catalytic converter, heated >500 °C) was used instead of dry synthetic air for pyrrole dilution to match and follow ambient humidity. Next, the catalytically scrubbed air was exchanged with ambient air, atmospheric OH reactive compounds entered the reactor, and competed with pyrrole for the available OH molecules. The resulting increase in pyrrole levels was detected via the PTR-MS as concentration level [C3]. These three monitored pyrrole levels were used to calculate the atmospheric total OH reactivity via the following equation.


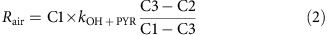


The reaction rate coefficient for pyrrole with OH has recently been determined in dependency of temperature and pressure by Dillon *et al*.^52^ According to the typical temperatures and pressure inside the glass reactor a value of *k*_OH+PYR_=1.2 × 10^−10^ cm^3^ molecule^−1^ s^−1^ was applied for analysis.

Typically the instrument was operated with initial pyrrole mixing ratios about 50–60 p.p.b.V. [C1] and OH mixing ratios of about 35–45 p.p.b.V. Errors in the detector (5%), rate coefficient (14%), gas standard (5%) and dilution (2%) lead to an overall uncertainty of 16%.

For the measurements from the vertical gradient system, the CRM was programmed to switch automatically between [C2] and [C3] in an hourly cycle (48+12 min). This allowed conducting total OH reactivity measurements for the first three gradients (each 16 min) and a background [C2] measurement for the last 12 min of the hour. During the [C2] measurements the gradient system switched between the different levels with a higher rate (1:30 min per height). The [C1] parts were measured regularly every other day.

For each measurement height 2 min averages were calculated from the 9 s PTR-MS raw data and the s.d. was given as uncertainty. The detection limit during the four Amazon rainforest measurement campaigns was about 3–4 s^−1^ (2σ of background [C2] noise). Throughout all campaigns the linear response of the CRM has been tested using an isoprene gas standard. The instrument showed excellent linearity over the wide range of tested OH reactivities and its response was close to 1 (Supplementary Fig. 8).

### Total OH reactivity data analysis

The procedures of data evaluation, corrections and analysis for the total OH reactivity measurements using CRM were previously described in detail^21^,^27^,^28^. Here we provide an overview of the total OH reactivity analysis for the presented Amazon rainforest observations.

*Humidity dependence of PTR-MS sensitivity*. The instrumental response of the PTR-MS to pyrrole is dependent on humidity^53^. Therefore separate calibrations for pyrrole in dry and humid conditions were performed. The dry calibration factor was applied to dry measurements, that is, during level [C1]. The wet calibration factor was applied to all wet measurements, that is, the [C2] and the [C3] parts. It has been observed that for relative humidity >30% the sensitivity of the PTR-MS to pyrrole did not vary. Since nitrogen is humidified for OH generation and the humid ambient sample is added to the mixture inside the glass reactor, our measurements during [C2] and [C3] always stay above this threshold, which allows working with one calibration factor for all humid parts.

*Humidity dependence of OH generation*. Although catalytically scrubbed air was used for the [C2] parts, humidity potentially varied during and between the [C2] and [C3] measurements. As the OH concentration inside the reactor strongly depends on the given humidity, a correction for these changes in the humidity was applied. For example, sections with higher humidity would have higher OH concentrations, and a smaller step between [C2] and [C3], hence the true total OH reactivity would be underestimated. A calibration with different known humidity levels in accordance to the PTR-MS water cluster (*m*/*z* 37) was performed and the data (during [C3]) accordingly corrected. This correction was especially critical, because the different levels of the 80 m profile experienced variable relative humidity.

The background [C2] was measured every hour, while the gradient system switched between the eight different levels. During that time, differences of the humidity at individual levels could be used as calibration since OH concentrations inside the CRM reactor varied accordingly. In extreme cases the humidity-dependent variability of OH concentration inside the CRM reactor caused a change in [C2] pyrrole levels of about 2 p.p.b.V. This was also the range of maximum [C2] drift over the time of the day that was mainly due to the ambient humidity variations.

*Accounting for not operating in pseudo-first-order conditions*. The calculation using the differences between the three pyrrole levels is based on the assumption of pseudo-first-order reaction conditions. This applies if pyrrole molecules are in excess in comparison to the generated OH. However, to provide detectable differences inside the reactor pyrrole and OH have been adjusted to the same order of magnitude (Pyr/OH≈1.5–2.5). A chemical model (Facsimile) helped to calculate theoretically expected pyrrole variations for the given experimental conditions. A comparison between the true total OH reactivity and the OH reactivity calculated using the model results within the above-mentioned equation provided a correction which was for the given conditions about 4–7% of the total measured OH reactivity.

*Dilution*. Finally, we had to account for the dilution of the sampled air. The addition of humidified nitrogen and pyrrole diluted the sample. The exact flow rates of all introduced gases have been monitored regularly and served to calculate a dilution factor.

*Possible interferences*. A known interference for the CRM is the recycling of OH within the reactor when high NO concentrations are apparent. Our instrument has shown that the reaction of ambient NO with the HO_2_ produced inside the reactor significantly impacts the results for total OH reactivity measurements when ambient NO levels are higher than 10 p.p.b.V. (ref. 27). However, during the Amazon rainforest measurement campaigns highest mixing ratios of NO reached about 1 p.p.b.V. at the soil level, hence no such interference was expected.

### Detection of VOC

The quadrupole PTR-MS(Ionicon, Austria) is capable of detecting gas-phase species with a higher proton affinity than water. This is the case for many VOC but not for molecular nitrogen, oxygen and argon. The molecules of interest are protonated through the reaction with H_3_O^+^, accelerated through a drift tube (2.2 mbar, 600 V, 127 Td) and their mass to charge ratio determined using a quadrupole mass spectrometer. Details about the measurement principal can be found elsewhere^29^,^54^.

The PTR-MS was calibrated for different humidity levels with a standard gas mixture (Apel-Riemer Environmental, 2013) for the following VOC: methanol (detected as protonated mass-to-charge ratio *m*/*z* 33), acetonitrile (*m*/*z* 42), acetaldehyde (*m*/*z* 45), acetone (*m*/*z* 59), dimethyl sulphide (DMS, *m*/*z* 63), isoprene (*m*/*z* 69), methyl vinyl ketone (MVK, *m*/*z* 71), methacrolein (MACR, *m*/*z* 71), methyl ethyl ketone (*m*/*z* 73), benzene (*m*/*z* 79), toluene (*m*/*z* 93), *o*-xylene (*m*/*z* 107), α-pinene (*m*/*z* 81 and *m*/*z* 137). For regular background measurements with VOC free air a catalytic converter (Platinum pellets, 400 °C) was automatically switched into the sampling line every hour.

The overall uncertainty of the VOC measurements was about 6–30%, the detection limit about 0.01–1 p.p.b.V., both strongly depending on the detected compound^29^.

## Additional information

**How to cite this article:** Nölscher, A. C. *et al*. Unexpected seasonality in quantity and composition of Amazon rainforest air reactivity. *Nat. Commun.* 7:10383 doi: 10.1038/ncomms10383 (2016).

## Supplementary Material

Supplementary Information Supplementary Figures 1-8, Supplementary Tables 1-2, Supplementary Notes 1-2 and Supplementary References.

## Figures and Tables

**Table 1:** Campaign average values and s.d.'s.

	**November 2012**	**March 2013**	**June 2013**	**September 2013**
*Total OH reactivity*				
0.05 m	(11.0±11.7) s^−1^	(7.4±4.7) s^−1^	(21.2±9.3) s^−1^	(38.4±28.3) s^−1^
24 m	(20.7±16.8) s^−1^	(9.9±5.2) s^−1^	(26.1±13.8) s^−1^	(62.4±41.3) s^−1^
79.3 m	(19.6±14.2) s^−1^	(11.3±6.5) s^−1^	(31.4±15.4) s^−1^	(60.2±37.8) s^−1^
Temperature (26 m)	(27.3±3.2) °C	(24.6±2.4) °C*	(25.4±2.5) °C	(26.8±3.4) °C
PAR (at noon)†	(1,433±744) μmol m^−2^ s^−1^	(929±620) μmol m^−2^ s^−1^	(1,170±690) μmol m^−2^ s^−1^	(1,775±670) μmol m^−2^ s^−1^
Rainday frequency	7.3	25.3	18.8	15.0

Calculated for the total OH reactivity, canopy temperatures and noon-time photosynthetically active radiation (PAR, 11:00–13:00 l.t.). In addition, the rainday frequency, the number of days with rain in the month of measurements are included. Details about the precipitation data for the four measurement intensives described here are given in Yañez-Serrano *et al*.^29^.

^*^No gradient measurements for temperature were available in March 2013. Instead the temperature monitored by a radiation sensor is given.

^†^Noon time (11:00–13:00 l.t.) PAR data were averaged over the course of the campaign.

**Table 2:** Correlations between total or missing OH reactivity and isoprene or MVK+MACR+ISOPOOH OH reactivity

**Total OH reactivity**		**Missing OH reactivity**		**Regimes of** **missing** **reactivity**
Isoprene OH reactivity				
y=(7.5±0.5)x+63.4	PCC=0.82	y=(6.1±0.4)x+60.2	PCC=0.76	High
y=(2.2±0.1)x+26.0	PCC=0.75	y=(1.0±0.1)x+23.6	PCC=0.46	Medium
y=(0.8±0.04)x+6.8	PCC=0.56	y=(−0.3±0.04)x+5.3	PCC=−0.27	Low
				
MVK+MACR+ISOPOOH OH reactivity				
y=(17.2±1.4)x+60.2	PCC=0.74	y=(13.7±1.4)x+58.4	PCC=0.69	High
y=(14.8±0.6)x+22.1	PCC=0.79	y=(8.5±0.6)x+19.6	PCC=0.62	Medium
y=(8.7±0.6)x+6.9	PCC=0.49	y=(−4.2±0.6)x+5.3	PCC=−0.27	Low

Linear fit parameters and PCC for correlations between total or missing OH reactivity to isoprene or isoprene products (MVK+MACR+ISOPOOH) OH reactivity as pictured in were calculated for high (dry season, >60 s^−1^), medium (dry season, <60 s^−1^) and low (wet season) missing OH reactivity regimes.
