# Supplementary material for: Unexpected seasonality in quantity and composition of Amazon rainforest air reactivity
Source: Nat Commun. 2016 Jan 22;7:10383. doi: 10.1038/ncomms10383 (PMC4735797; doi:10.1038/ncomms10383)
Supplement: Supplementary Information — Supplementary Figures 1-8, Supplementary Tables 1-2, Supplementary Notes 1-2 and Supplementary References. [file ncomms10383-s1.pdf]

Supplementary Figure 1: Experimental set-up at ATTO site.

80 m walk-up tower embedded in the rainforest with eight inlet lines for vertical gradient measurements (left). View on the inlets mounted at 0.05 m, 0.5 m, 4 m and 24 m (from bottom to top) with details of soil, leaf litter, understory, and heterogeneous top of the canopy (right).

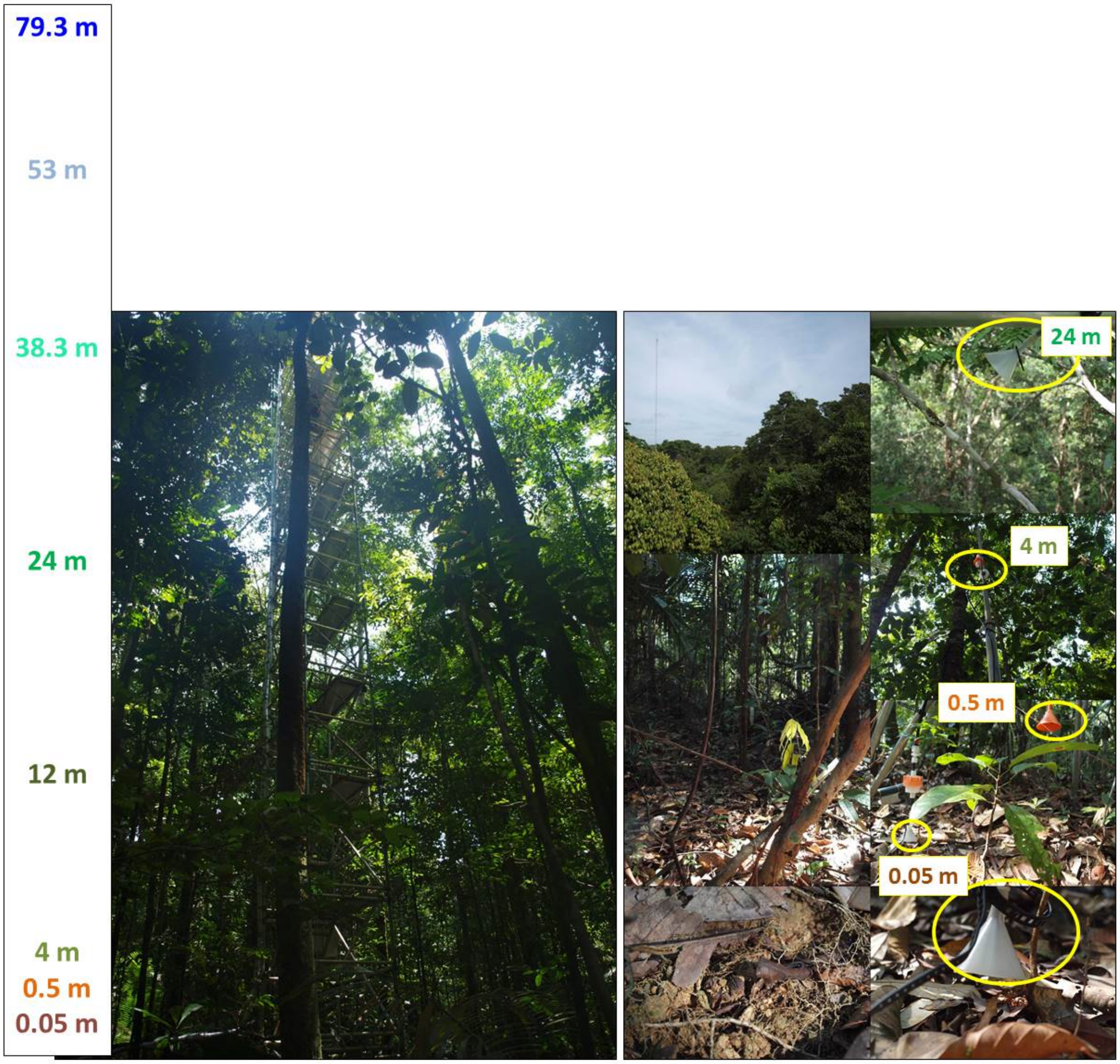

Supplementary Figure 2: Observed total OH reactivity, temperature and photosynthetically active radiation during four intensive field measurement campaigns.

Total OH reactivity was measured alternating in 2 minute intervals for eight heights between 0.05 m to 80 m. Three heights are pictured to present the soil level (0.05 m), canopy (24 m) and highest level in the atmosphere (79.3 m). These data are presented for all four campaigns in November 2012, March 2013, June 2013 and September 2013. During two campaigns we performed a test with a separate inlet line at 12 m (2.-5. and 22.-23. November 2012) and 24 m (16.-19. and 21. September 2013) and found no significant difference between the individual inlet and the gradient inlets.

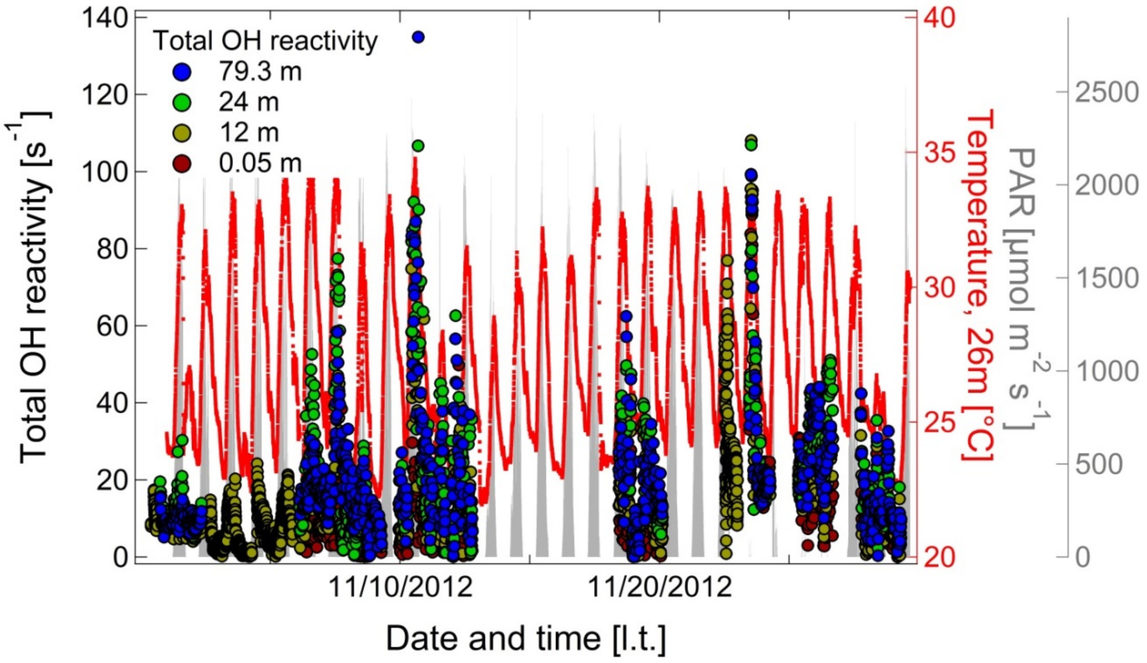

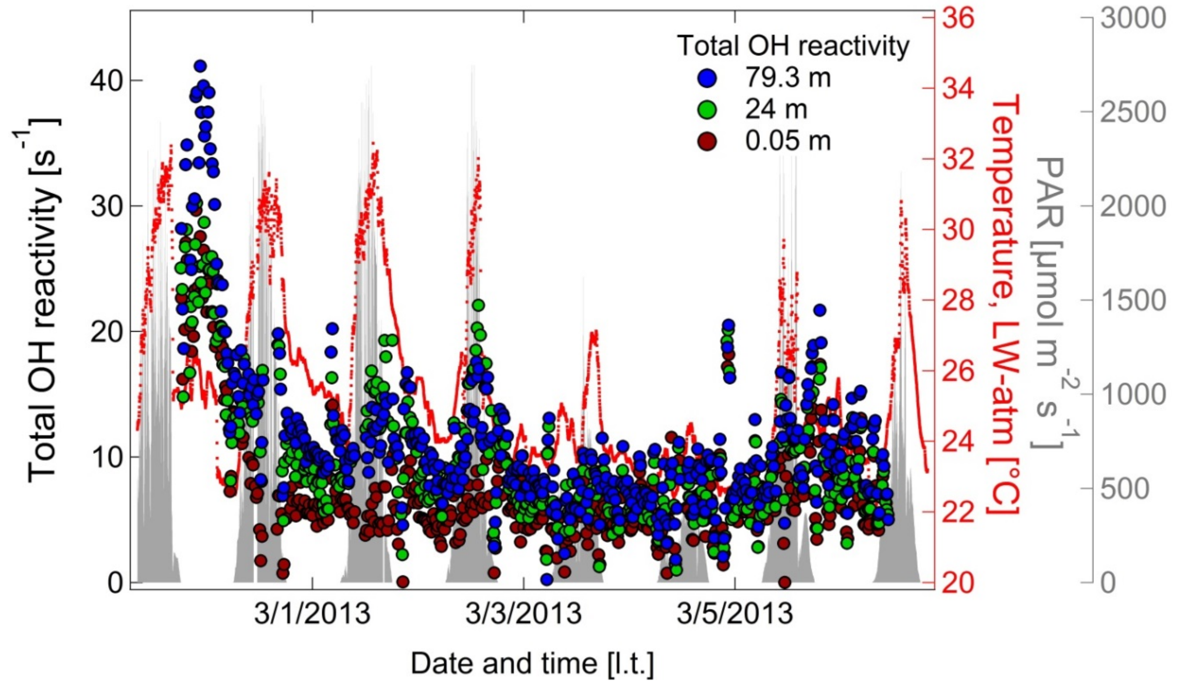

Total OH reactivity
79.3 m
24 m
0.05 m
Total OH reactivity [s -1]
Temperature, LW-atm [°C]
PAR [μmol m -2 s -1]
3/1/2013
3/3/2013
3/5/2013
Date and time [l.t.]

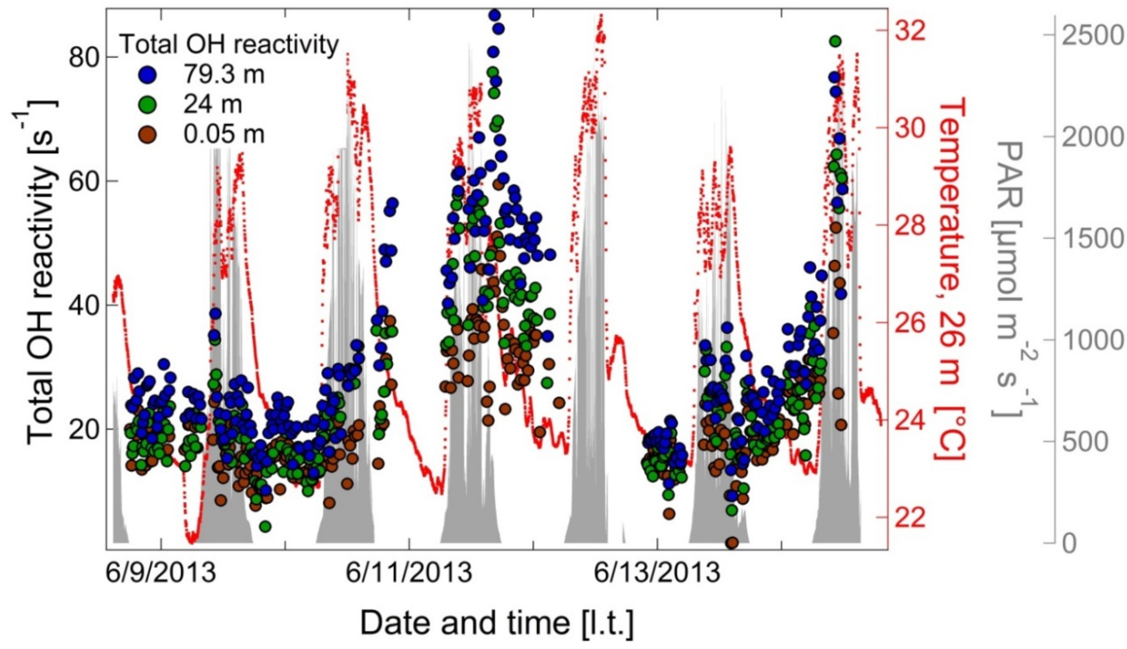

Total OH reactivity
79.3 m
24 m
0.05 m
Total OH reactivity [s -1]
Temperature, 26 m [°C]
PAR [μmol m -2 s -1]
6/9/2013
6/11/2013
6/13/2013
Date and time [l.t.]

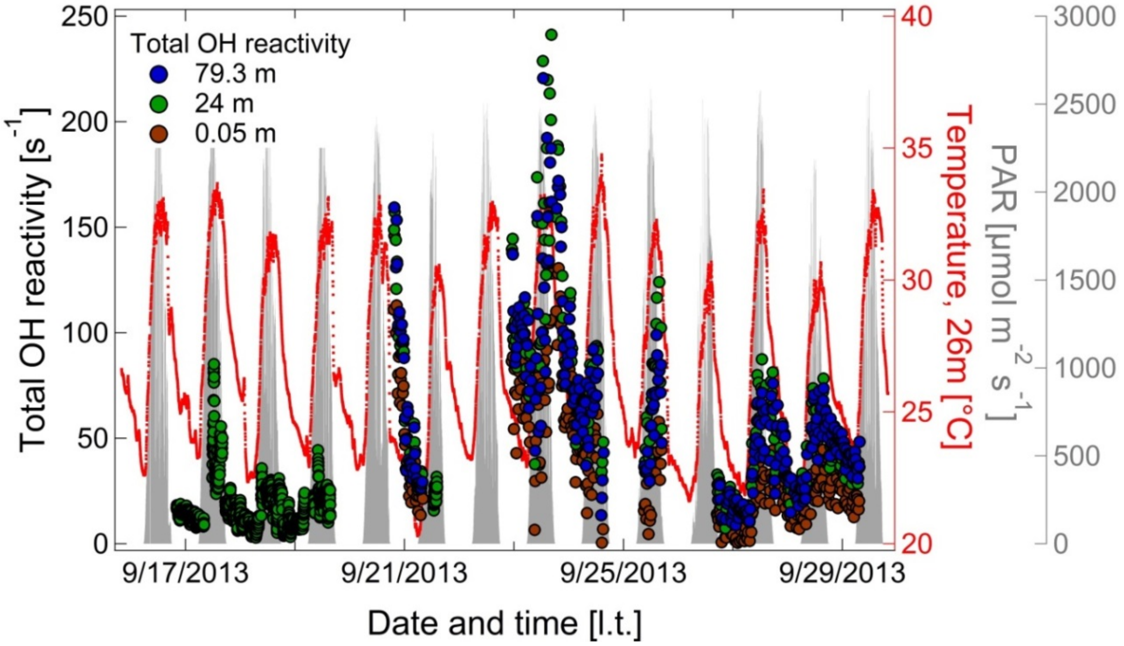

Total OH reactivity
79.3 m
24 m
0.05 m
Total OH reactivity [s-1]
Temperature, 26m [°C]
PAR [μmol m-2 s-1]
Date and time [l.t.]
9/17/2013
9/21/2013
9/25/2013
9/29/2013

Supplementary Figure 3: Soil temperature and moisture in dry and wet season.

Soil temperature was measured in 3 depths (0.1 m, 0.2 m, 0.4 m) and soil water content monitored in 6 depths (0.1 m, 0.2 m, 0.3 m, 0.4 m, 0.6 m, 1 m) during the dry-season and wet-season measurement campaign in September and March 2013.

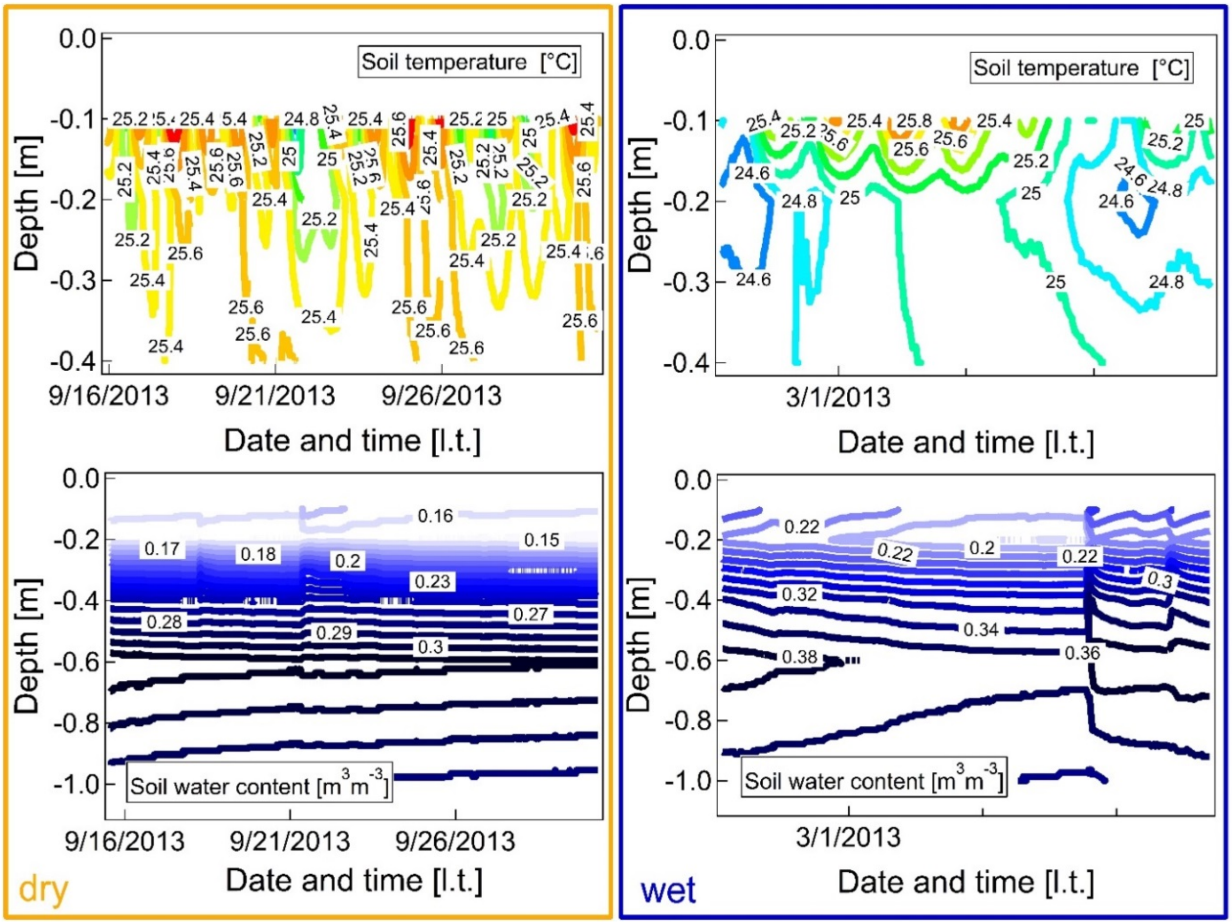

Supplementary Figure 4: Calculation of gradients above the canopy.

Gradients above the canopy (24 to 79.3 m) have been calculated as simple linear regression between the measured total OH reactivity (or isoprene or MVK+MACR+ISOPOOH OH reactivity) and the height above the canopy. Supplementary Figure 4 explains the principal calculation: A linear regression curve was calculated for the four levels above the canopy (24 m, 38.3 m, 53 m and 79.3 m) and the observed OH reactivity with respect to the highest measurement level. Therefore the 79.3 m level was associated to 0 m, the 53 m level to 26.3 m (difference to top level), the 38.3 m level to 41 m and the 24 m level to 55.3 m. The linear regression slope is positive for high values inside the canopy, and negative for high values at the top level. Please note that this simple approach does not reflect ecosystem fluxes. It helps to define and compare the direction of vertical gradients for OH reactivity.

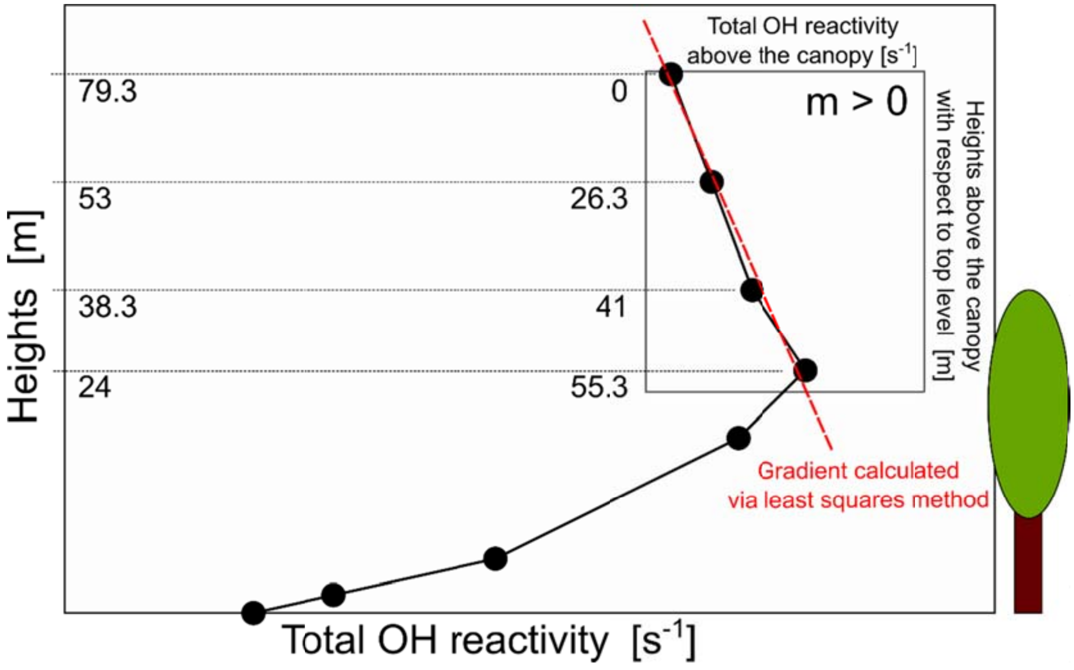

Supplementary Figure 5: GC-FID (and GC-MS) monoterpene analysis for Amazon rainforest samples during different seasons and heights.

The relative contribution of each monoterpene is given as percentage of the entire sum of all monoterpenes.

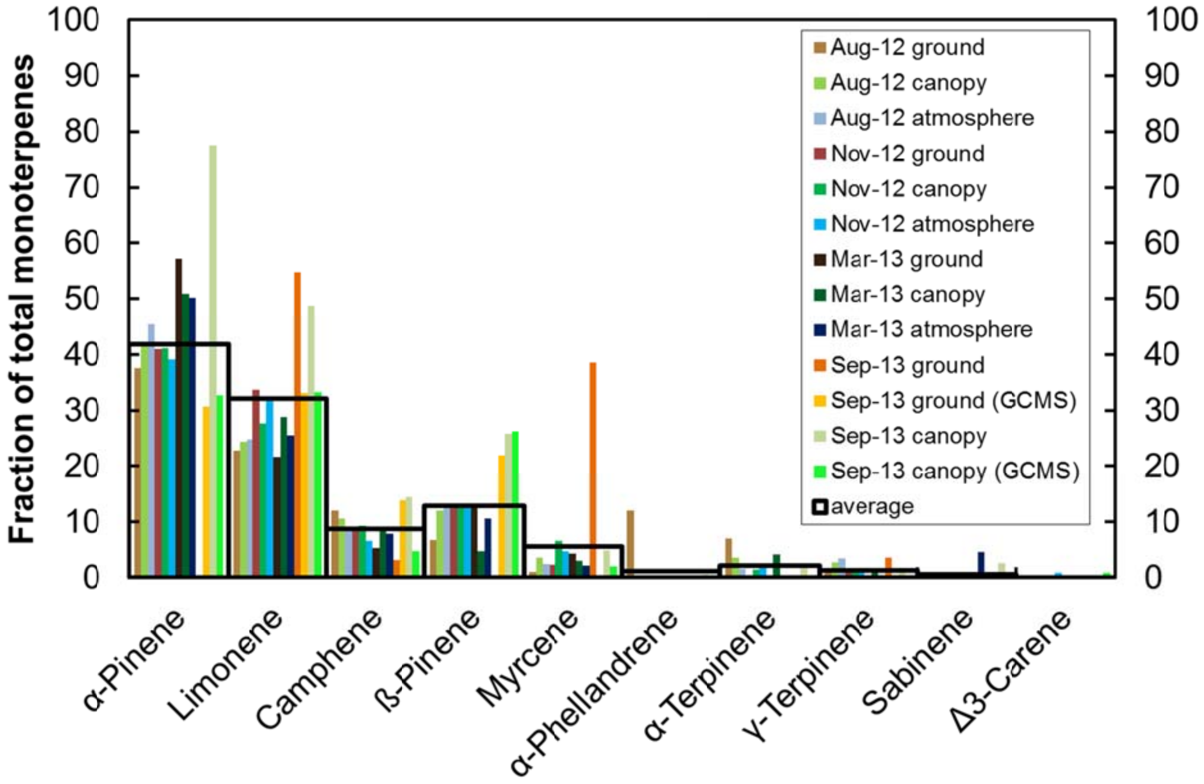

Supplementary Figure 6: Correlations of total and missing OH reactivity with isoprene and MVK+MACR+ISOPOOH OH reactivity.

All four campaigns data-sets are included in this figure for three different heights (soil level=0.05 m, canopy=24 m, highest level above canopy=79.3 m). Total and missing OH reactivity show generally a positive correlation with the measured direct emissions (isoprene) and atmospheric isoprene photooxidation products (MVK+MACR+ISOPOOH). However, this correlation is not very significant and seems to consist of various chunks of data with different strong correlations.

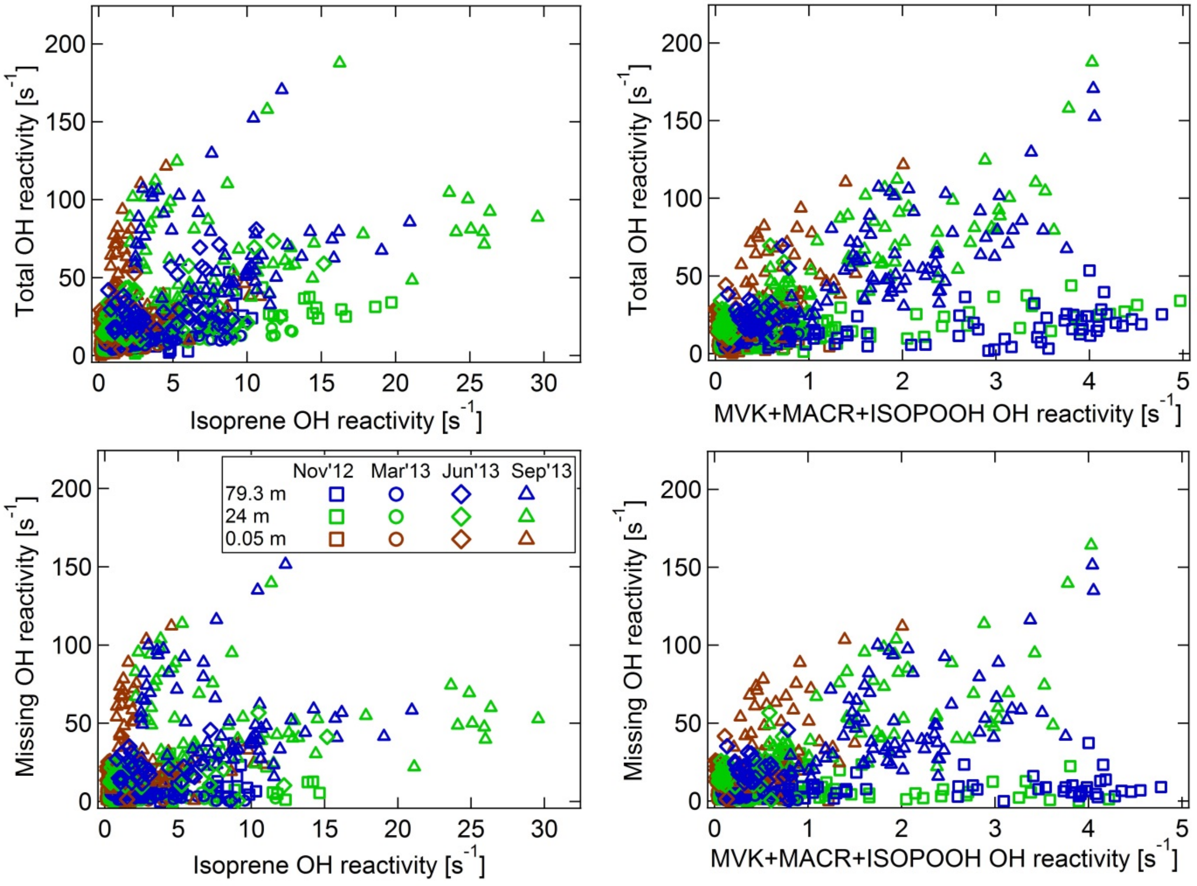

Supplementary Figure 7: Residual OH reactivity for night (00-03l.t.) and day (12-15l.t.) during three regimes of low, medium and high missing OH reactivity.

The residual OH reactivity is the difference between observed missing OH reactivity and empirical model which combines typical vertical distributions of photooxidation products (MVK+MACR+ISOPOOH) and biogenic emissions (isoprene). Interestingly, for all cases the residual is positive close to the ground pointing towards a missing OH sink other than isoprene-like tree emissions or isoprene products-like atmospheric photooxidation products.

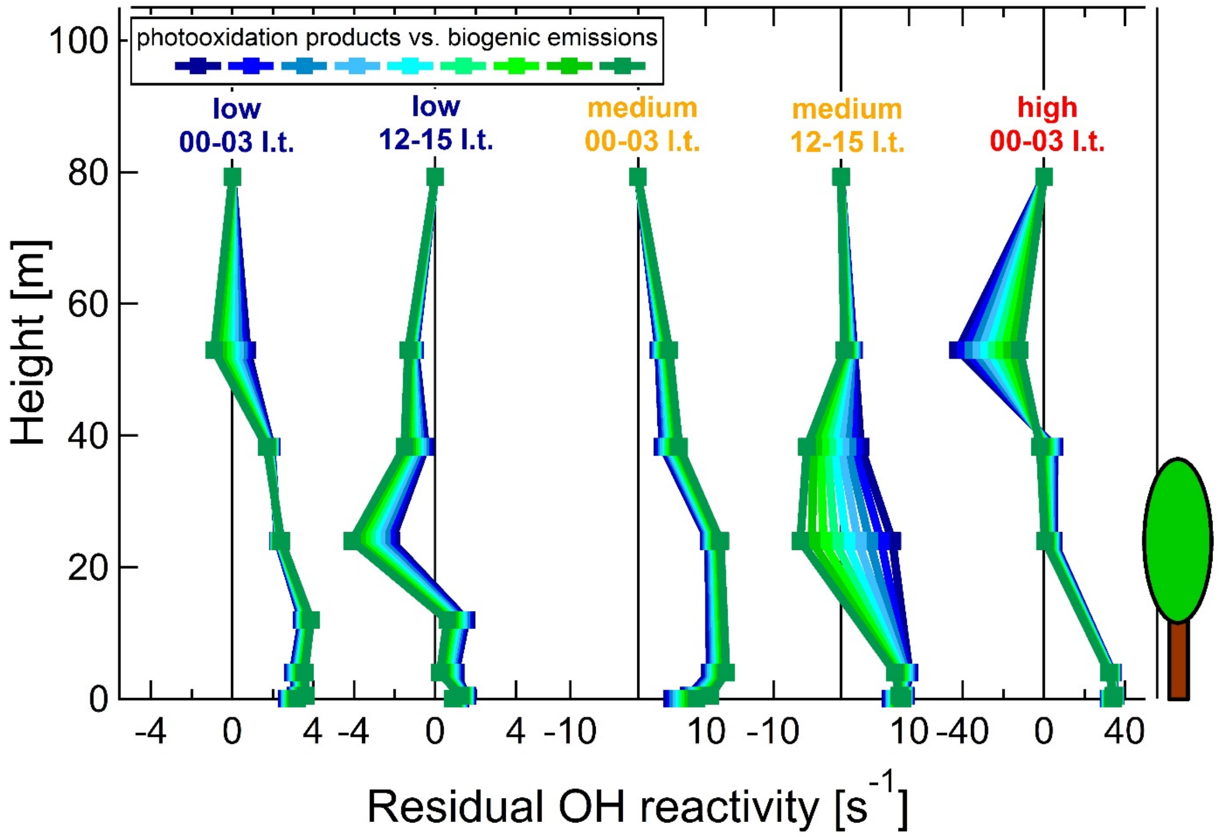

Supplementary Figure 8: Tests for linear response with isoprene-standard.

As a quality check known amounts of isoprene have been injected into the CRM instead of an atmospheric sample. The linear response was good over a wide range of reactivities and stayed stable with time.

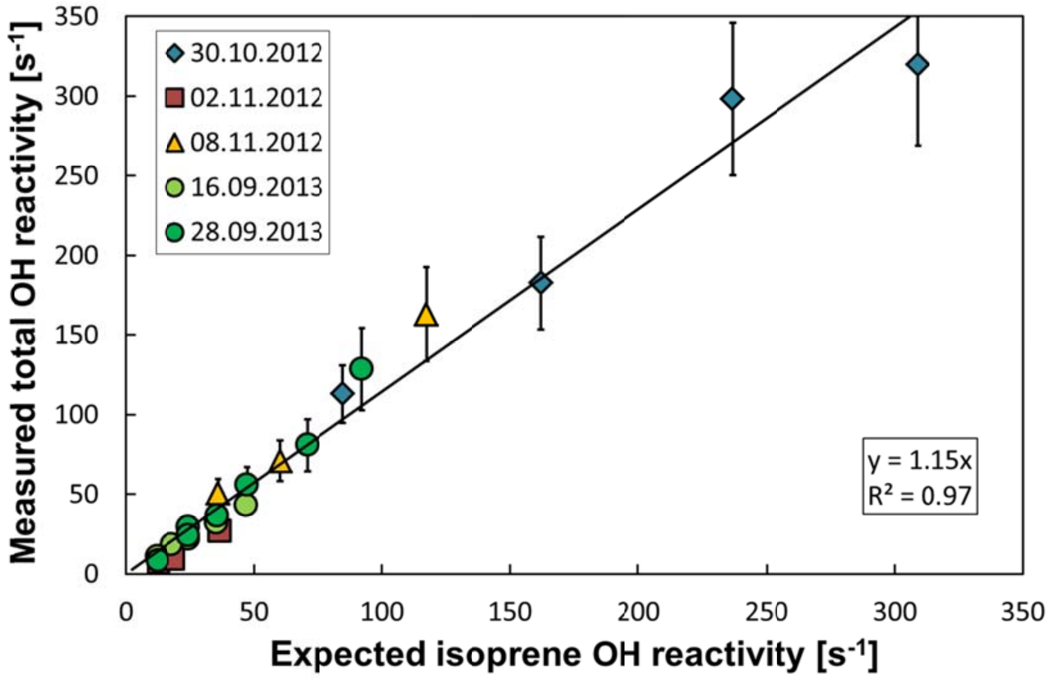

Supplementary Table 1: Individually measured contributors to total OH reactivity.

Single compound reaction coefficients for oxidation with OH (in $cm^3 molecule^{-1} s^{-1}$) and campaign averaged OH reactivities (in s^{-1}) at 24 m inside the canopy.

		dry season		wet season	
**compound**	***k*** **[$cm^3 molec^{-1} s^{-1}$]**	**average [s^{-1}]**	**standard deviation**	**average [s^{-1}]**	**standard deviation**
methanol	9.0×10^{-13}	0.072	0.037	0.014	0.015
acetonitrile	2.2×10^{-14}	0.000094	0.000034	0.000031	0.000017
acetaldehyde	1.5×10^{-11}	0.27	0.072	0.26	0.30
acetone	1.8×10^{-13}	0.0069	0.0023	0.0025	0.0022
isoprene	1.0×10^{-10}	8.7	7.5	2.4	3.0
MVK+MACR+ISOPOOH	5.62×10^{-11}	1.74	1.04	0.14	0.17
MEK	1.2×10^{-12}	0.010	0.005	0.0053	0.0062
monoterpenes	1.17×10^{-11}	0.158	0.084	0	0
benzene	1.2×10^{-12}	0.0025	0.0013	0	0
toluene	5.6×10^{-12}	0.101	0.045	0.028	0.051
CO	1.44×10^{-13}	0.51	-	0.22	0.03
NO	3.3×10^{-11}	0.81	-	0.81	-
NO_2	1.1×10^{-11}	0.081	-	0.081	-
O_3	7.3×10^{-14}	0.027	-	0.016	-
methane	6.4×10^{-15}	0.3	-	0.3	-

Supplementary Table 2: Empirical model calculations to account for the observed missing OH reactivity at 79.3 m during dry season for times of medium missing reactivity.

For night and day, multiples of the observed isoprene and isoprene products (MVK+MACR+ISOPOOH) OH reactivities have been summed to explain the missing OH reactivity in 79.3 m. The resulting model vertical profiles of OH reactivity are compared to the observations in Figure 7.

	**Medium missing OH reactivity (79.3 m)**	**Isoprene OH reactivity**	**MVK+MACR+ISOPOOH OH reactivity**	
Nighttime	25.1 s^{-1}	2.2 s^{-1}	1.1 s^{-1}	
		**Multiplication factor**		**Ratio**
		1.15	20.83	10:90
		2.30	18.51	20:80
		3.45	16.20	30:70
		4.60	13.89	40:60
		5.75	11.57	50:50
		6.90	9.26	60:40
		8.05	6.94	70:30
		9.20	4.63	80:20
		10.35	2.31	90:10
Daytime	41.0 s^{-1}	10.5 s^{-1}	2.4 s^{-1}	
		**Multiplication factor**		**Ratio**
		0.39	15.67	10:90
		0.78	13.93	20:80
		1.17	12.19	30:70
		1.56	10.45	40:60
		1.95	8.70	50:50
		2.34	6.96	60:40
		2.73	5.22	70:30
		3.12	3.48	80:20
		3.51	1.74	90:10

Supplementary Note 1: Total OH reactivity budget.

Parallel to the total OH reactivity, several VOC (methanol, acetonitrile, acetaldehyde, acetone, isoprene, isoprene products, MEK, monoterpenes, benzene, toluene) were detected with a second PTR-MS[1]. Although typical mass scans performed by the PTR-MS in the rainforest boundary layer[2] do show multiple additional minor signals beyond those used here for the reactivity budget analysis, these signals were neither unequivocally identified or calibrated and thus could not be used in this budget. At the same tower additionally ambient levels of CO, NO, NO_2, O_3, and CH_4 were monitored. The OH reactivity of each individual sink compound could be calculated as product of concentration and reaction rate coefficient. Supplementary Table 1 summarizes all measured OH sinks, their IUPAC recommended reaction rate coefficient (at 25°C, if available) and the average OH reactivity calculated for dry- and wet-season measurement campaigns.

In the dry-season canopy isoprene is the greatest instantaneous OH sink with an absolute average OH reactivity of 8.7 s^{-1}. This is on average 13.5% of the measured total OH reactivity. Measured isoprene products were the second most important OH sink and accounted for on average 2.7% of the total OH reactivity measured inside the dry-season canopy. Each of the other measured individual OH sinks contributed on average to less than 2% of the measured dry-season total OH reactivity in 24 m.

CO, NO, NO_2, O_3 and CH_4 are part of the continuous observations at the ATTO site. However, due to incomplete data-sets during the intensive campaigns constant OH reactivities have been calculated in order to maximize the comparable data for calculated and measured total OH reactivity. Maximum values for NO and NO_2 were about 1 ppbV and 0.3 ppbV (close to the soil) which correspond to an OH reactivity of 0.812 s^{-1} and 0.081 s^{-1}, respectively. In the wet-season OH reactivities of CO and O_3 were calculated from observations shortly before the field

campaign. In the dry-season CO measurements were available in parallel to the total OH reactivity observations and only ozone OH reactivities had to be estimated from previous measurements. However, especially ozone is not very reactive and the contribution to the total calculated OH reactivity low (e.g. 0.3% of the average isoprene OH reactivity, dry-season at 24 m). Ambient methane levels usually ranged from 1.85 ppmV to 1.95 ppmV. Although methane existed in much higher mixing ratios than all other OH sinks, it is not very reactive and its contribution to the overall OH sink was relatively low (e.g. 3% of the average isoprene OH reactivity, dry-season at 24 m).

It should be noted that on the PTR-MS protonated mass-to-charge ratio m/z=71 potentially three important isoprene oxidation products are monitored. The instrument was calibrated for the isoprene products methyl vinyl ketone (MVK) and methacrolein (MACR). However, in the Amazon rainforest environment also isoprene hydroxyl hydroperoxides (ISOPOOH) are likely to be detected on the same mass[3,4]. Since with our set-up we cannot differentiate between the different isoprene oxidation products, we assume that the PTR-MS detects all with the same sensitivity. Also, in order to determine a rate coefficient for OH reactivity calculation we assume that the observed PTR-MS mixing ratio in ambient air is equally due to MVK, MACR and ISOPOOH. With $k_{OH+MVK}=2.9\times10^{-11}$ $cm^3 molecule^{-1} s^{-1}$, $k_{OH+MACR}=3.07\times10^{-11}$ $cm^3 molecule^{-1} s^{-1}$ (recommended IUPAC values), $k_{OH+ISOPBOOH}=5\times10^{-11}$ $cm^3 molecule^{-1} s^{-1}$, and $k_{OH+ISOPDOOH}=1.15\times10^{-10}$ $cm^3 molecule^{-1} s^{-1}$ (MCM v3.3, via website: http://mcm.leeds.ac.uk/MCM) an average rate coefficient of $k_{OH+MVK+MACR+ISOPOOH}=5.62\times10^{-11}$ $cm^3 molecule^{-1} s^{-1}$ was calculated.

The PTR-MS was not capable of separating the various monoterpenes and detected them as sum at the same mass. However, the OH reactivity of different monoterpenes can vary over

magnitudes[5]. That is why for the total OH reactivity calculation it is important to define the composition of the detected total monoterpene signal. Details about the rainforest monoterpene composition have been determined via samples on absorbent tubes (Carbograph I, Carbograph V) that were collected in the field in parallel to total OH reactivity and PTR-MS measurements and analyzed offline in the laboratory via a Gas Chromatograph-Flame Ionization Detector (GC-FID)[1]. Supplementary Figure 5 presents all available monoterpene data that were collected and analyzed from August 2012 to September 2013. Slight variations between different seasons and heights are apparent, however a general trend is visible. On average throughout all samples the most dominant monoterpenes were α-pinene (42%), limonene (32%) and β-pinene (13%). The weighted average of all detected monoterpene rate coefficients led to the estimate of a reaction coefficient for the total monoterpenes with OH of $k_{OH+MT}=1.17\times10^{-11}$ $cm^3molecule^{-1}s^{-1}$. During the dry-season (September 2013) monoterpenes accounted for an average OH sink as strong as 0.158 s^{-1}. During the wet-season their signal was below the detection limit of the PTR-MS instrument. Besides speciation of the monoterpenes for the OH reactivity assessment several additional identified species were detected by the GC-FID (including pentane, butanal, 2-butanone, hexane, pentanal, heptane, hexanal, octane, heptanal, and nonane) and amounted to maximum OH reactivity of 0.91 s^{-1} in the dry season. This data was not included in the calculated OH reactivity budget due to the lack of continuous observations.

Supplementary Note 2: Vertical gradients of missing OH reactivity and empirical model.

Whether the nature of the observed missing OH reactivity was more like biogenic forest emissions or atmospheric photooxidation products was examined by using the typical tree primary emission and typical atmospheric photooxidation products profiles of the observed isoprene and isoprene products (MVK+MACR+ISOPOOH). As an upper limit it was assumed that all of the missing OH reactivity detected in 79.3 m is due to the sum of unmeasured biogenic emissions and undetected photooxidation products. Therefore the typical vertical profile of isoprene was used as a biogenic emission proxy and the typical vertical profile of MVK+MACR+ISOPOOH (m/z 71) was used to present photooxidation products. Multiples of those experimentally observed profiles were added to cover different compositions from photooxidation products dominated to biogenic emissions dominated. In the first extreme case 90% of the missing OH reactivity in 79.3 m was assumed to be due to compounds that follow a typical products vertical profile and only 10% of the mission OH reactivity was assumed to be due to unmeasured isoprene-like compounds. For the other extreme case, the 79.3 m missing OH reactivity was assumed to consist to 90% of primary biogenic emissions and only to 10% of photooxidation products. While the composition was varied in 9 steps from one to the other extreme case, the sum of both typical OH sink profiles could be observed to emerge from a profile that is shaped like photooxidation products to a profile that is shaped like a biogenic tree emission.

Supplementary Table 2 presents for the medium missing reactivity during dry-season, how the missing OH reactivity in 79.3 m was accounted for by different variations of summed isoprene and MVK+MACR+ISOPOOH OH reactivities.

Supplementary References

1. Yañez-Serrano, A.M. et al., Diel and seasonal changes of biogenic volatile organic compounds within and above an Amazonian rainforest, *Atmos. Chem. Phys.* **15,** 3359-3378 (2015).
2. Williams, J. et al., An Atmospheric Chemistry Interpretation of Mass Scans Obtained from a Proton Transfer Mass Spectrometer Flown over the Tropical Rainforest of Surinam, *J. Atmos. Chem.* **38**, 133-166 (2001).
3. Liu, Y.J., Herdlinger-Blatt, I., McKinney, K.A., Martin, S.T. ,Production of methyl vinyl ketone and methacrolein via the hydroperoxyl pathway of isoprene photooxidation, *Atmos. Chem. Phys.* **13**, 5715-5730 (2013).
4. Rivera-Rios**,** J. C. et al., Conversion of hydroperoxides to carbonyls in field and laboratory instrumentation: Observational bias in diagnosing pristine versus anthropogenically controlled atmospheric chemistry, *Geophys. Res. Lett.* 41 (2014).Atkinson, R., Arey, J., Atmospheric Degradation of Volatile Organic Compounds, *Chem. Rev.* **103**, 4605-4638 (2003).
5. Atkinson, R., Arey, J., Atmospheric Degradation of Volatile Organic Compounds, *Chem. Rev.* **103**, 4605-4638 (2003).
